# Remanufacturing Perovskite Solar Cells and Modules–A Holistic Case Study

**DOI:** 10.1021/acssusresmgt.3c00042

**Published:** 2024-01-31

**Authors:** Dmitry Bogachuk, Peter van der Windt, Lukas Wagner, David Martineau, Stephanie Narbey, Anand Verma, Jaekeun Lim, Salma Zouhair, Markus Kohlstädt, Andreas Hinsch, Samuel D. Stranks, Uli Würfel, Stefan W. Glunz

**Affiliations:** † Fraunhofer Institute for Solar Energy Systems ISE, Heidenhofstr. 2, 79110 Freiburg, Germany; ‡ Energy21 BV, Orteliuslaan 893, 3528 BR Utrecht, The Netherlands; § Solar Energy Conversion Group, Department of Physics, Philipps-University Marburg, Renthof 7, 35032 Marburg, Germany; ∥ Solaronix SA, Rue de l’Ouriette 129, 1170 Aubonne, Switzerland; ⊥ Institute of Chemical Sciences and Engineering, École Polytechnique Fédérale de Lausanne (EPFL), 1951 Sion, Switzerland; ¶ ERCMN FSTT Abdelmalek Essaadi University, Av. Khenifra, 93000 Tétouan Morocco; △ Freiburg Materials Research Center FMF, University of Freiburg, Stefan-Meier-Str. 21, 79104 Freiburg, Germany; & Department of Chemical Engineering & Biotechnology, University of Cambridge, Philippa Fawcett Drive, Cambridge CB3 0AS, United Kingdom; % Department for Sustainable Systems Engineering (INATECH), University of Freiburg, Emmy-Noether-Straße 2, 79110 Freiburg, Germany

**Keywords:** perovskite, remanufacturing, photovoltaics, sustainability, LCA, recyclilng

## Abstract

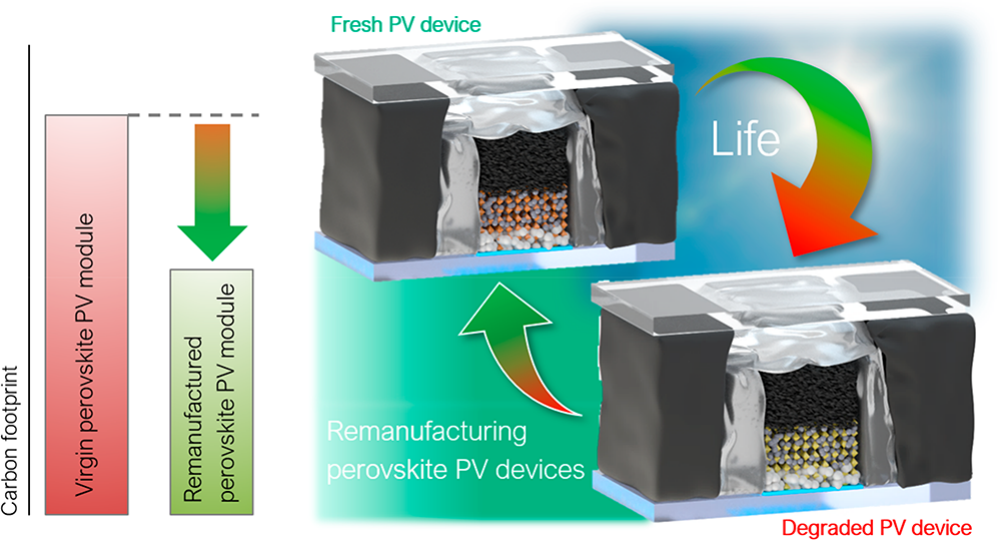

While perovskite photovoltaic (PV) devices are on the verge of commercialization, promising methods to recycle or remanufacture fully encapsulated perovskite solar cells (PSCs) and modules are still missing. Through a detailed life-cycle assessment shown in this work, we identify that the majority of the greenhouse gas emissions can be reduced by re-using the glass substrate and parts of the PV cells. Based on these analytical findings, we develop a novel thermally assisted mechanochemical approach to remove the encapsulants, the electrode, and the perovskite absorber, allowing reuse of most of the device constituents for remanufacturing PSCs, which recovered nearly 90% of their initial performance. Notably, this is the first experimental demonstration of remanufacturing PSCs with an encapsulant and an edge-seal, which are necessary for commercial perovskite solar modules. This approach distinguishes itself from the “traditional” recycling methods previously demonstrated in perovskite literature by allowing direct reuse of bulk materials with high environmental impact. Thus, such a remanufacturing strategy becomes even more favorable than recycling, and it allows us to save up to 33% of the module’s global warming potential. Remarkably, this process most likely can be universally applied to other PSC architectures, particularly n-i-p-based architectures that rely on inorganic metal oxide layers deposited on glass substrates. Finally, we demonstrate that the CO_2_-footprint of these remanufactured devices can become less than 30 g/kWh, which is the value for state-of-the-art c-Si PV modules, and can even reach 15 g/kWh assuming a similar lifetime.

## Abstract 2

A novel thermally assisted mechanochemical approach is developed to remanufacture fully encapsulated perovskite solar cells (PSCs), allowing most of the device constituents to be re-used and saving up to 33% of the module’s global warming potential.

## Introduction

To comply with the climate goals of the Paris Agreement, the global installed photovoltaic (PV) capacity is projected to enter the multi-terawatt-scale in the forthcoming years.^1−4^ Despite PV’s potential to be an environmentally friendly alternative to fossil fuel energy sources, emissions associated with PV are non-negligible,^4,5^ and the projected increase in PV installations is estimated to lead to 78 million tons of waste cumulatively by 2050.^6^ Recycling could mitigate the amount of PV-related waste and has become one of the central discussions in the PV sector.^7−9^

However, recent studies have shown that bulk recycling is not necessarily more environmentally friendly than technologically simpler End-of-Life (EoL) treatments such as landfill disposal or incineration.^10^ This is due to the energy-intensive techniques used to process EoL PV module materials into usable parts for recycled modules, e.g., grinding (into culets), separation, and remelting of glass. The re-use of entire modules or parts thereof could offer an environmentally friendly alternative to such methods. This could be particularly viable for PV modules compatible with solution-based processing techniques such as perovskites. Here, the active components of the solar cell can potentially be washed off to directly re-use bulk materials with high environmental impact such as glass. When a combination of re-used, recycled, repaired, or replaced parts serves to manufacture a new product, the term remanufacturing can be used. Throughout this paper, this term is employed to refer to a combination of processes.

Perovskite photoabsorbers have a high absorption coefficient, thereby requiring significantly less material to build a PV module in comparison to silicon-based devices.^11^ In addition to their potential compatibility with recycling, perovskite solar cells (PSCs) thus require fewer materials and can possibly reduce PV-associated waste streams. Moreover, PSCs typically rely on earth-abundant materials^12^ and should not face issues with resource scarcity. Due to this combination of factors, PSCs could offer a more sustainable alternative to silicon PV, while solution-processed PSCs are also compatible with high throughput and cheap manufacturing.^13^

PSCs face a number of challenges that currently limit industrial-scale production. This mostly concerns stability issues, resulting in low lifetimes, and inhomogeneous deposition, which limit the power conversion efficiency (PCE) on larger substrates.^14^ While current large research efforts are dedicated to overcome these issues, methods for perovskite recycling or remanufacturing should likewise be considered and evaluated before eventual commercial production, as such assessments can potentially guide the development of technologies in the direction of high recyclability and low environmental impact.^15,16^

Several works have demonstrated recycling approaches of PSCs, showing almost no performance loss, even after multiple recycling cycles.^17,18^ To our knowledge, however, previously published reports only developed processes for recycling PV cells, and no real case studies on encapsulated devices have been performed. Yet, to produce modules with lifetimes suitable for actual commercial deployment (>20 years), perovskite solar modules (PSMs) need to be equipped with additional barriers to limit environmental degradation (e.g., from moisture and air), such as encapsulation materials and a back sheet glass.^5,19^ Ideally, these should also be complemented by lead-sequestrating materials embedded into the modules.^20−22^ Thus, practical applications for recycling unencapsulated PSCs are limited without suitable methods to cleanly get rid of the encapsulant material.

Similarly, life cycle assessments (LCAs) have been conducted on PSCs before. As commercial PSM production currently does not exist, its environmental impacts cannot yet be accurately measured or compared to incumbent PV technologies such as silicon or cadmium telluride (CdTe). In order to approximate such a comparison, the production process of an early-stage technology (in this case, PSMs) is often forecasted to represent a commercial stage.^15^

Previous LCAs on perovskites, however, typically assessed the environmental impacts of PSCs only based on lab-scale production energy and material input data (often, linear extrapolation is used to account for the inefficient use of machinery). This is the case for studies that researched PSC production processes incompatible with scaling up (e.g., spin coating),^23,24^ but it also persists in more recent LCAs that reviewed scalable manufacturing processes.^25−27^ Due to the low efficiency of lab-scale processes, LCAs that use such data may not accurately predict the environmental impact of PSMs produced on a larger scale. Additionally, previous works often did not include the environmental impacts of balance of system (BOS) and balance of module (BOM) materials, such as inverters and encapsulation, even though this is prescribed by the IEA PVPS PV LCA guidelines.^28^ This further limits the accuracy of these LCAs and possible comparisons with other PV technologies.

In this work, we demonstrate for the first time a remanufacturing strategy for glass–glass encapsulated perovskite solar cells.^29−33^ Our study presents a facile experimental method to remove the edge-sealant, encapsulant, back electrode, and degraded perovskite, allowing reuse of the device constituents with the highest global warming potential–the glass substrate and back-sheet. Through a prospective LCA, we approximate the carbon footprint of a commercially produced PSM and evaluate the environmental footprint associated with the developed remanufacturing strategy. We show that the global warming potential of PSMs (and other types of PV) can be reduced substantially by directly re-using the glass substrate after EoL, and that this method is favored over traditional forms of PV recycling. Although in this study we utilize PSCs with carbon-based electrodes (CPSCs) due to their promising commercialization potential and device stability, the developed remanufacturing approach herein is also applicable to other PSC architectures. This remarkable strategy paves a new way to strongly reduce the global warming potential of PV modules and substantially reduce their waste streams, which is essential for terawatt-scale PV installations of the future.

## Results and Discussion

### Environmental Potential of Recycling and Remanufacturing Encapsulated Perovskite PV Devices

From an environmental perspective, the focus of any waste reduction route should be to recover and re-use those materials with high associated environmental impacts. To identify these “environmental hotspots” of perovskite PV modules via life-cycle assessment, the device architecture must be first specified.

**Figure 1:**
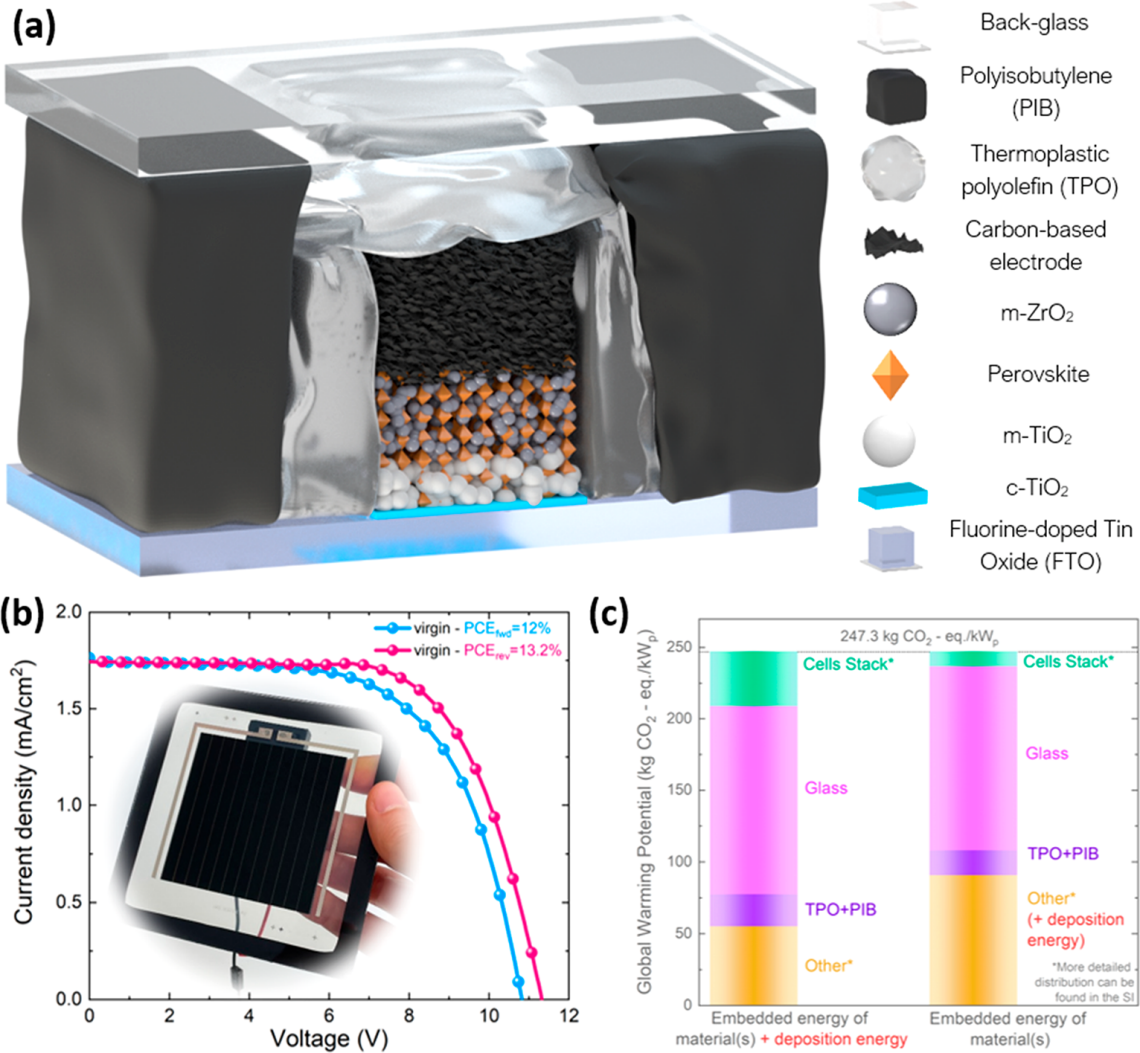
(a) Illustration of device stack of the encapsulated photovoltaic perovskite solar cells and modules with carbon-based electrodes employed in this study, with photographs (right) of the cells prior to and after the encapsulation with TPO and PIB. (b) JV-characteristics of a perovskite solar module encapsulated with TPO and PIB shown in the photograph having a PCE of 13.2% and 12% in reverse and forward scans, respectively. (c) Global warming potential obtained from an LCA of a module based on the device structure shown in (a). Both bars show the same total GWP, but the left bar itemizes the impact of the fabrication of each module constituent whereas the right bar shows the distribution of the GWP by the materials of the constituents.

Here, we look at PSMs with carbon-based electrodes constituted by abundant materials that can also be deposited by processing techniques compatible with commercial production. Specifically, the solar cell stack of the considered modules includes a fluorine-doped tin oxide (FTO) front electrode, compact and mesoporous titanium dioxide (c-TiO_2_ and m-TiO_2_, respectively), zirconium dioxide (ZrO_2_), and a carbon-based back electrode (Figure 1a). This device architecture omits materials with high costs, is compatible with fast deposition processes, and has shown high device stability^34^ compared to other architectures, giving it a high potential for commercialization. After infiltration and crystallization of the perovskite inside the mesoscopic scaffold, the devices are encapsulated with thermoplastic olefins (TPO), polyisobutylene (PIB)-based edge seal, and a back glass sheet. The justification of the encapsulation method selection can be found in Supplementary Note 1.

In order to quantify the environmental profile of such modules, the PCE has to be known. In contrast to most LCAs on perovskite devices found in literature, where PCE is assumed based on reported small (<1 cm^2^) cell efficiencies, we manufactured encapsulated perovskite solar modules based on the stack shown in Figure 1a. The active area of the perovskite solar modules with carbon-based electrodes (CPSMs) was 56.7 cm^2^ with a geometrical fill factor (gFF) of 93%. According to the current–voltage (*IV)* measurements (Figure 1b), our perovskite solar modules have a PCE of 13.2%, which is the highest PCE of modules with carbon-based electrodes of comparable size (Figure S1) according to our knowledge. Hence, we use this value for our LCA, details of which can be found in the Supplementary Note 2.

Although we plotted the entire environmental profile of the module (Figure S3), we focus primarily on the global warming potential (GWP), expressed in kg of CO_2_-equivalent per kW_p_ of generated power, in this work. Figure 1c presents two distribution charts of individual contributions of device constituents to the total GWP. The bar on the left side shows the amount of kg of CO_2_-eq emitted during the full processing of each module component (including the energy needed to deposit the layers, e.g., for screen-printing and sintering). Thus, this graph shows the potential GWP reduction per layer that can be achieved if layers are re-used without re-processing after EoL. The chart on the right side visualizes the embedded GWP per deployed material itself and has a separate part included in the “Other” contribution to account for all the energy and solvents used to fully process the layers (and overhead energy). More detailed distribution of individual contributions (e.g., cell layers, junction box, bus bars) to the total GWP (and other environmental impact categories) can be found in Figures S4 and S5. We highlight that during this LCA, we deliberately made conservative assumptions (such as a total glass thickness of 5.2 mm, which can be further reduced, and conservative estimates of overhead electricity), to avoid portraying an ideal case and showcase a more realistic scenario of the potential GWP upon commercial production. Nevertheless, we note that the total GWP of encapsulated CPSMs was estimated to be 247.3 kg of CO_2_-eq/kW_p_, which is one of the lowest GWPs reported for perovskite PV modules (Figure S6), according to our knowledge. The LCAs of perovskite PV devices in the literature often linearly extrapolate the known values of energy consumption of lab-scale production to manufacturing processes of ∼m^2^ modules. This gives rise to an assumption of energy-inefficient production and an overestimation of the required resources for large-scale PSM production. Additionally, the PSCs assessed in previous LCAs often contain metal electrodes, which are deposited using energy-intensive evaporation techniques.^35,50^ Consequently, such devices have a substantially higher GWP than perovskite devices with carbon electrodes (Figure S22).

The second chart depicts that only a small part of the GWP contribution of the cell stack comes from the materials itself (5%), while the majority originates from the energy and solvents used to deposit these layers. Thus, for the cell stack, possible GWP reduction can primarily be achieved by re-using the components in layer form (as they were originally deposited), which should be the initial aim of PSM remanufacturing schemes. Contrarily, the GWP mitigation potential for the recovery and subsequent reprocessing of the raw materials found in cell stack materials is limited.

For the recycling part of the LCA, we do not consider module components such as the junction box, as these are typically separated before PV module recycling/remanufacturing and sent to designated electronic waste recycling plants.^36^ Besides the materials and energy used directly for the fabrication of PSMs, GWP impact comes from indirect processes such as overhead electricity, infrastructure, and packaging (included in “Other” in Figure 1c), which are not module components that can be recycled. Recycling the TPO and the PIB-based edge seal is relatively complicated because they both contain additives (necessary to obtain desirable encapsulation traits). The presence of such additives may lead to degradation during melting and re-extrusion or cause catalyst deactivation in thermal recycling processes, thereby reducing the quality of the material.^37^ High-quality recycling of the encapsulant and edge seal would require processes and installations outside the scope of this project and is thus not considered here.

Both charts clearly demonstrate that the main GWP reduction can be achieved by re-using or recycling the glass (front and back), which can potentially reduce the GWP of PSMs by up to 53% and 52%, respectively. However, glass recycling comes with additional transport and energy to produce glass cullet and subsequently melt this into recycled glass. Due to these added efforts, glass recycling would likely bring little to no environmental impact reduction with respect to GWP (Figures S7 and S8). Correspondingly, our main aim was to design a remanufacturing process that allows the direct re-use of glass and as many PSC layers as possible, since this can potentially provide a substantial decrease in GWP impact.

As mentioned, hybrid halide perovskites are compatible with a wide range of suitable charge selective layers and electrodes processable using different techniques. This could potentially lead to a large variation in the GWP impact (and recycling hotspots) between PSMs with different architectures. However, the results from Figure 1c show that the photovoltaic cell constituents (and their embedded energy) have a relatively small contribution to the overall carbon footprint of a PSM, which mostly comes from the glass substrate and back-sheet (used for all PSMs that do not have a flexible substrate). The contribution of the cell layers becomes even smaller when the whole PV system is considered, as BOS parts also make up a considerable amount of the carbon footprint per produced kWh (over half of the GWP at higher lifetimes, Figure S21).

Additionally, we show that the GWP of the module does not change much when other, similar, PSC architectures are used (between 11% less to 30% more kg of CO_2_-eq/m^2^ for the cell stack of carbon-based PSMs, Figures S22 and S23) or when alternative materials or solvents are used for the perovskite layer (Figures S24 and S25). The only exception to this is the substitution of the carbon electrode by a (thermally evaporated) silver one (Figure S23), which increases the GWP (per m^2^) of the cell stack over 5-fold and would result in an almost 60% higher total module GWP (without BOS), assuming the same PCE. The additional GWP is mostly caused by the high electricity use of thermal evaporation (the material impact of silver is minimal). As such, this deposition method should be avoided if the aim is to produce PSMs with a low GWP (and overall environmental impact), unless the electricity use of thermal evaporation can be substantially reduced.

Within the boundaries of solution processing, however, the GWP impact presented here should accurately predict that of PSMs with various other cell architectures. Regardless of the deposition method, the glass substrate will contribute considerably to the overall GWP of PSMs and we expect that the proposed remanufacturing method can be applied to reduce the environmental footprint of most perovskite (and potentially other) PV devices on glass substrates.

### Development of the Resource Separation Process

To develop such a remanufacturing technique, we manufactured individual perovskite solar cells with carbon-based electrodes (CPSCs) with an active area of ∼1.5 cm^2^. Statistics of the device power conversion efficiency (PCE) shown in Figure S8 demonstrate high reproducibility of this encapsulation approach with an average PCE (in reverse-scan) of 14.5% and 13.7% before and after encapsulation, respectively. We also note an increase in the level of hysteresis upon encapsulation. The reduction in PCE and increase in hysteresis could be attributed to a slight decomposition of methylammonium-rich perovskite used in these CPSCs, which tend to be particularly sensitive to elevated temperatures that were reached during the encapsulation process (cells were encapsulated at 110 °C for 10 mins). Similar effects upon encapsulation of such type of cells have been previously observed in literature.^51^

Among all the cell components (presented in Figure 1a), perovskite is the one most likely to deteriorate in the shortest time, meaning that in order to replace it, at least the PIB, TPO, and back-glass have to be removed. Given that PIB is a rubber elastomer with glass transition temperature below room temperature, additional heating induces softening, allowing an easier mechanical separation of the back-sheet glass from the FTO (with cell stack). However, we note that heating the encapsulated perovskite above 160 °C would melt the TPO due to the presence of polypropylene (with melting point at 160 °C), which then strongly adheres to the FTO surface. Therefore, we identified the ideal processing window for glass substrate separation (FTO and back-glass) to be between 120–140 °C. The separation was assisted by making an incision through the PIB rubber with a blade, which can also be done with a thin metallic wire on larger scale, since the gap between the glass substrates is around 400–500 μm, as can be seen from the SEM image in Figure S9. After separation (Figure S10) the glass substrates were left to cool down to room temperature before the encapsulant removal step.

**Figure 2:**
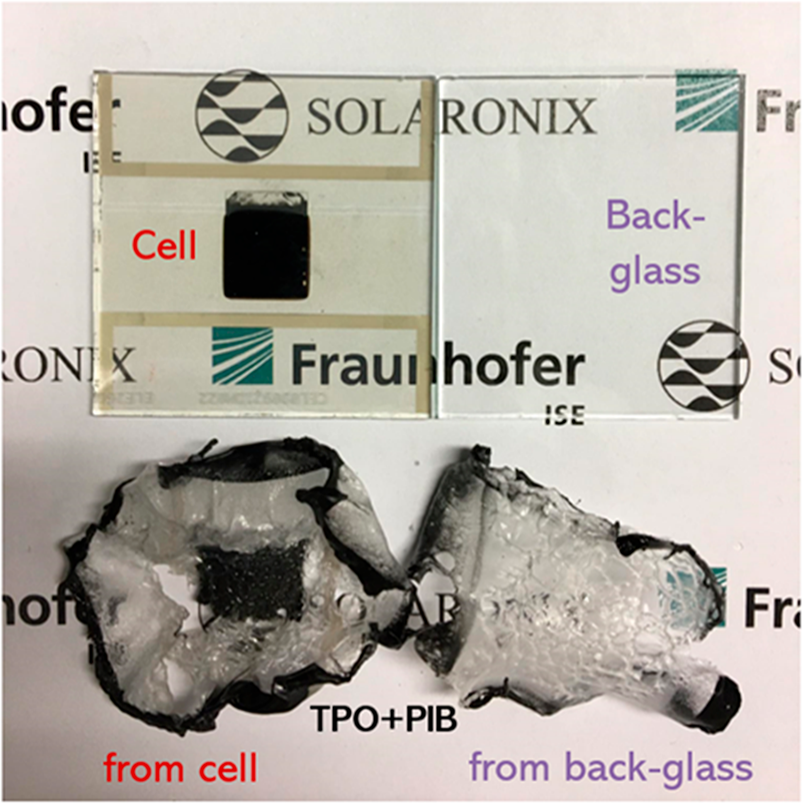
Photographs demonstrating a neat removal of encapsulants (TPO + PIB) and back-glass from the solar cell using the proposed thermally assisted mechanochemical process, allowing recycling of them to potentially reduce the device GWP.

From the screened solvents, which could potentially dissolve PIB and TPO, none of them allowed a complete removal without an intensive additional manual effort (Figure S11). Therefore, a “one-step” recycling process using a chemical bath with solvent that can dissolve edge-seal, encapsulant, and perovskite was not possible for devices encapsulated with TPO and PIB. However, after keeping the substrates with PIB and TPO in acetone for 1 h, both the edge sealant and the encapsulant could be easily peeled off (Figure 2). Although acetone itself does not dissolve any of the cell layers except perovskite, keeping the cell stack with TPO in acetone longer results in partial decomposition of the carbon with perovskite (Figure S12). The exact composition of the TPO materials can vary, depending on the manufacturer, but typically their main components are various types of polyethylene (PE).^38^ Since PE can be dissolved in acetone, the loss of TPO adhesion to the FTO is attributed to the partial dissolution of PE in TPO.

**Figure 3:**
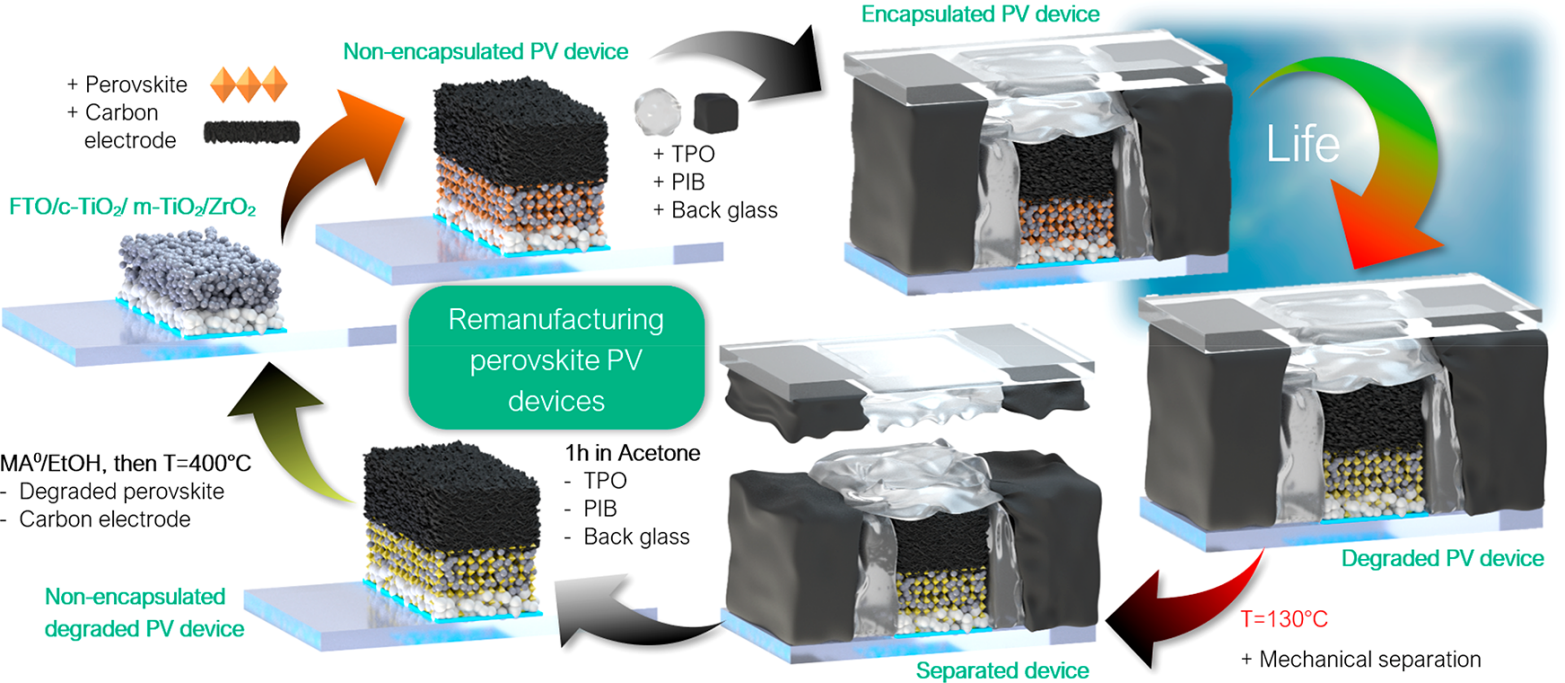
Proposed remanufacturing route for carbon-based perovskite PV modules based on a thermally assisted mechanochemical method to separate the back-glass, encapsulants, degraded perovskite, and carbon layer. This allows to re-use the rest of the layers and to lower the GWP of a perovskite PV module significantly.

After the TPO and PIB were peeled off, the devices were dipped into a bath of methylamine (MA^0^) and ethanol to liquefy and wash out the perovskite.^39^ This solvent was selected for this remanufacturing approach because it has the lowest boiling point among the commonly used solvents (DMF, DMSO, NMP, GBL, MA^0^/EtOH) allowing for its rapid evaporation from the cell stack, after perovskite has been removed.^40^ Further annealing at 400 °C allows removal of perovskite and carbon remnants from the stack, leaving m-TiO_2_ and ZrO_2_ intact. Cross-sectional SEM images in Figure S13 demonstrate a comparison between these layers which were “as-deposited” (before the device manufacturing is complete) and the same layers after TPO, PIB, carbon, and perovskite have been removed, clearly showing that the layer morphology and thickness are preserved. Therefore, carbon deposition and perovskite solution infiltration, followed by the encapsulation with TPO and PIB can be performed again to complete the remanufacturing loop, as depicted in Figure 3. This remanufacturing route is an appealing form of EoL treatment due to its simplicity, effective re-use of most of the layers in the cell stack, and lack of energy-intensive process steps, such as glass melting.

The recovery of carbon or perovskite was not considered since their recycling might be more challenging in terms of processing, translating into additional GWP and reducing the environmental benefit. Removal of perovskite and its recovery at high purity to allow for recrystallization in a solar cell might become particularly challenging, since its optoelectronic quality must be high to ensure decent device performance. Although methylamine liquefaction and recrystallization has been demonstrated for PSC recycling before, this process requires high amounts of methylamine to remove perovskite to a reasonable extent, which typically results in creation of pin-holes and defects,^39,41^ reducing its environmental performance due to lower PCE and stability. Additionally, the amount of perovskite that can be recovered per square meter is rather small (∼20 g/m^2^) and has a relatively low contribution to the total environmental impact of the module. Thus, the potential environmental benefit from re-using perovskite will quickly be offset by the environmental impact related to solvents and energy used in the process. We note, however, that purification of these recovered materials can potentially be carried out in large, specialized factories, so that their GWP could be lower than that of primary materials. However, the question of whether such specialized factories will exist and how much GWP could potentially be reduced is beyond the scope of this work. Considering that TPO and PIB are nearly intact after their removal from the module, these could possibly be recycled to further reduce PV material waste. However, as mentioned, the quality of such materials may deteriorate after recycling, which makes them less attractive for PV applications (the quality of encapsulant materials is very important for the lifetime of modules). Hence, recycled TPO and PIB would likely be more suitable to produce (lower quality) materials for other purposes.

### Solar Cell Remanufacturing

**Figure 4:**
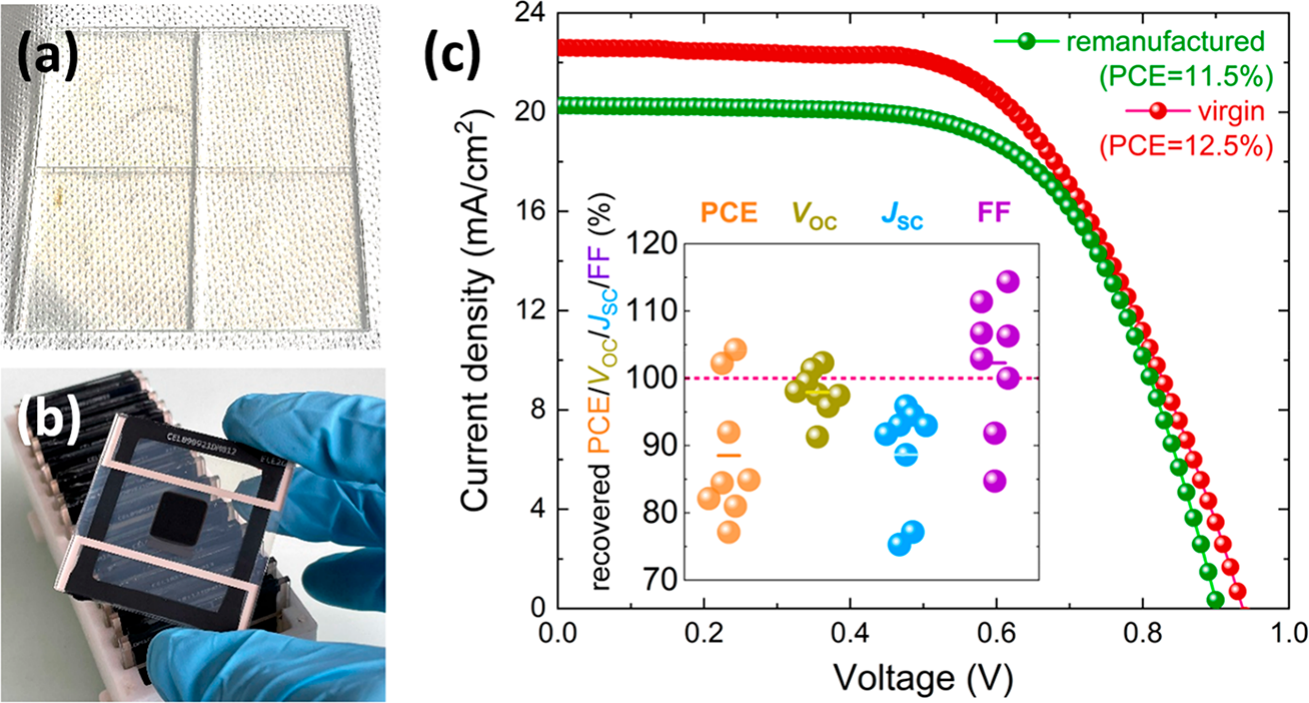
Photographs of recovered (a) FTO/c-TiO_2_/m-TiO_2_/ZrO_2_ and (b) complete encapsulated CPSCs. (c) JV-curves of a representative CPSC before and after recycling with 92% recovered PCE. Device statistics are shown in the inset, demonstrating a change in each individual JV-parameter: PCE, *V*_OC_, *J*_SC_, and FF after recycling relative to the initial (virgin) characteristics.

The encapsulated CPSCs were remanufactured according to the developed thermally assisted mechanochemical approach. The removal of perovskite and carbon, according to the procedure in Figure3, allows reuse of the metal oxide layers (TiO_2_, ZrO_2_) deposited on the FTO (Figure 4a) in order to remanufacture encapsulated CPSCs. After remanufacturing, the devices look pristine without obvious damage to the cell active area (Figure 4b). To evaluate the efficacy of our proposed remanufacturing route, we compared the JV-parameters of cells before and after remanufacturing. Figure 4c demonstrates that while some remanufactured cells have a slightly better PCE than before, the average recovered PCE after remanufacturing is 88% of their initial value. We note that all the cells (virgin and recycled) were always measured 4 days after encapsulation (stored in the dark under 30–40% RH, room temperature).

The PCE loss occurs primarily due to a significant decrease in *J*_SC,_ suggesting the presence of additional optical or charge extraction losses. Considering that, visually, the FTO substrates with metal oxide layers appeared similarly to the virgin ones, a strong reduction in photocurrent is not expected. Therefore, we speculate that the surface of the oxide layers could have been modified after the remanufacturing procedure, leaving traces of decomposed perovskite, which causes additional charge-extraction losses. However, further analysis of these losses and their mitigation could be addressed in a following work. The JV-parameters obtained from forward scans can be found in Figure S14.

While the proposed remanufacturing approach was successfully implemented in the CPSCs shown above, we must note that these cells were not degraded. In order to see if the procedure can still be implemented in degraded CPSCs, we subjected them to 1,000 h of continuous light-soaking under open-circuit and to a water permeation test, in which the encapsulated cells were also submerged in water for 1,000 h (Figure S15). As evident from the photographs in Figure S16, the cells still retained a dark appearance, confirming the presence of the photoactive perovskite layer and strong degradation inhibition by the demonstrated encapsulation method. Nevertheless, we exposed the degraded solar cells (Figure S17a) to the MA^0^ liquefying solution and an additional heat-treatment to see if the procedure is also applicable in this case. Photographs in Figure S17b clearly confirm that the proposed approach can also remove the degraded layers while keeping the porous oxide layers intact, similarly to the results shown earlier.

## Effect of the Proposed Approach

The proposed remanufacturing method distinguishes itself from traditional PV (bulk) recycling methods, as most of the device constituents are simply re-used and only perovskite, electrode, encapsulant, and edge-seal need to be replaced. As can be seen from Table 1, these constituents account for only around 15% of the device GWP, while the re-used components represent over 62% of the module GWP (note that some of the GWP, such as from overhead electricity or packaging, is not directly embedded in the module).

**Table 1:** List of the Device Constituents, Their EoL, and Corresponding GWP

	Re-used?	GWP (kg CO_2_-eq/kW_p_)	% of total GWP
Glass	yes	131.3	53.1
FTO	yes	9.4	3.8
c-TiO_2_	yes	3.6	1.5
m-TiO_2_	yes	5.6	2.2
ZrO_2_	yes	4.5	1.8
Perovskite	no	8.2	3.3
Carbon	no	7	2.8
TPO + PIB	no	22.4	9

**Figure 5:**
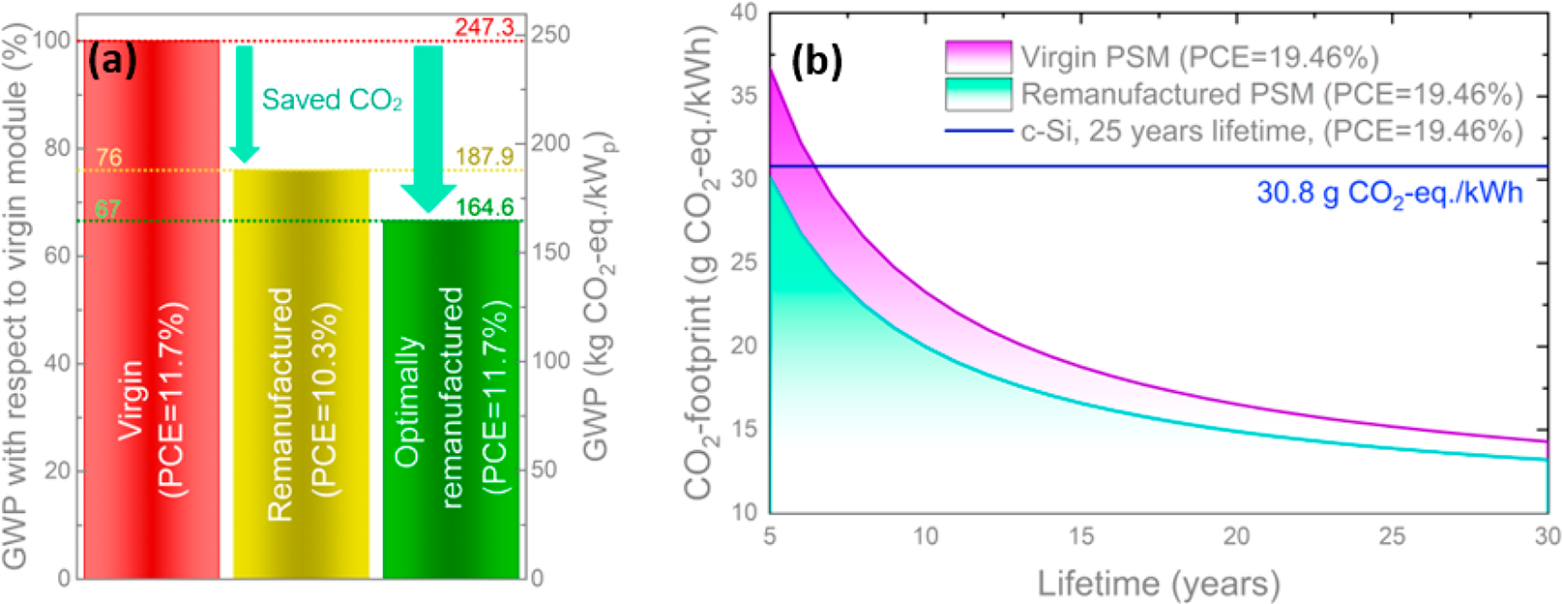
(a) GWP comparison of the virgin CPSMs in comparison to the recycled device with a loss in PCE equivalent to the mean experimentally obtained value of 12% relative PCE loss in Figure 4a, as well as a hypothetical case of no performance loss. (b) CO_2_-footprint of virgin and remanufactured PSMs as a function of device lifetime in comparison to c-Si modules^42^ currently available on the market (as baseline). Note that in both graphs the PCE over the total area is considered (i.e., a product of PCE on the active are and geometrical FF).

Next, we evaluate the environmental benefit of remanufacturing modules (1 m^2^) using our newly developed thermally assisted mechanochemical approach. A comparison in GWP for virgin, remanufactured with 88% of recovered PCE (based on the mean performance loss of cells shown in Figure 4c), and “optimally remanufactured” (without any loss of PCE after remanufacturing) is presented in Figure 5a. A complete environmental profile of the remanufactured modules is shown in Figure S18. Our findings illustrate how this remanufacturing route could reduce the GWP of CPSMs by 24% or even by 33% if the remanufacturing process is optimized. This could result in 82 kg of CO_2_-eq/kW_p_ savings as well as an environmental impact reduction in most other categories (Figure S18). Note that, despite fully re-using parts constituting 62.4% of the GWP, the reduction in GWP (compared to virgin modules) due to remanufacturing is substantially lower. This is because of the additional processes required for remanufacturing. Predominantly the chemical treatment in MA^0^/EtOH and acetone baths, but also the temperature-assisted mechanical separation of glass substrates and annealing at 400 °C result in non-negligible CO_2_-eq emissions.

In Figure S19, we observe that, with our assumptions, such as linear degradation over lifetime, location of Freiburg, Germany, with an annual generation of 1429.2 kWh/m^2^/y (more details are discussed in the SI), a lifetime of over 16 years (Figure S19) is required for virgin PSMs to have lower CO_2_ emissions per kWh than c-Si modules at −30.8 g CO_2_-eq/kWh^42^ (background parameters adjusted to match those used in this study, see SI). However, this “break-even” lifetime requirement can be decreased to 10.7 years when the modules are remanufactured. With longer lifetime, the impact of the layers and solvent recycling becomes almost negligible, minimizing the GWP impact to that of the FTO-glass and the Balance of System (BoS). This is due to the relatively higher impact of BoS components at higher lifespans because the lifetimes of these parts are independent of that of PSMs. Correspondingly, we recommend further research into the efficient reuse and remanufacturing of BoS components to decrease the carbon footprint of not only perovskite but also PV power generation as a whole.

Despite the diminishing results of remanufacturing at increased lifetimes, Figure 5 shows that module-related emissions of perovskite PV can be substantially reduced by remanufacturing, especially when the PCE loss is kept to a minimum. Additionally, the remanufacturing process described here can likely still be optimized to further reduce the GWP. For example, we did not consider solvent recycling or re-use (either directly or through distillation) during the remanufacturing process. This was left out of scope due to a lack of data on the energy requirements of industrial solvent recovery of specific solvents, while using generic solvent recovery data comes with a high uncertainty as the energy required to recover solvents can vary widely per solvent^43,44^ Still, solvent recycling could likely further reduce the GWP of remanufactured PSMs, given that the demand for solvent purity in the described process is rather low and solvents comprise the bulk of the GWP of remanufacturing efforts (Figure S17).

It is likely that upon eventual market introduction, the PCEs of PSMs will approach those of c-Si devices (rooftop perovskite PV systems with a PCE of 16 to 18% and a lifetime of 20 years are estimated to have an LCOE of around 0.10 €/kWh,^45,46^ adjusted for Freiburg irradiation levels, which is at the upper end of the spectrum for rooftop c-Si PV systems in Germany^47^). Figure 5b presents the CO_2_-footprint of highly efficient virgin and remanufactured PSMs having the same PCE as the state-of-the-art c-Si modules, clearly showing that a further decrease in carbon emissions per kWh could be attained with the proposed production and remanufacturing schemes. If such high-performing PSMs were to be produced similarly and remanufactured according to the procedure we describe, their CO_2_-footprint would already be lower than that of c-Si modules even after a lifetime of 5 years. With similar lifetimes (25 years), these modules would have a footprint of only 14.4 g of CO_2_-eq/kWh—less than half of c-Si.

Even though we conducted experiments on PSCs with only the device architecture outlined in this study, we note that the presented remanufacturing strategy utilizes solvents and treatments that do not decompose inorganic device constituents, such as metal oxide layers. Thus, this approach can also be applied to a broad range of n-i-p devices (which currently still hold the last certified PCE world-records among PSCs^48^) and some inorganic p-i-n based ones, for which we expect similar reductions in GWP.

## Conclusion

In summary, this holistic study presents a life-cycle assessment of perovskite solar modules and how their remanufacturing may affect their carbon footprint. After identifying the glass substrate as the main contributing factor to the global warming potential of PSMs, we developed an effective approach to re-use the substrate together with most of the cell layers and replace the missing components, thereby developing a perovskite PV device remanufacturing process for the first time. This thermally assisted mechanochemical remanufacturing method consists of only a few process steps, does not use toxic solvents such as DMF, and produces remanufactured encapsulated devices with a PCE close to 90% of virgin devices. Moreover, this procedure is universally applicable to other perovskite-based devices as well, especially n-i-p-based devices, which utilize inorganic metal oxide layers deposited on glass substrates. Despite the obvious need for perovskite PV devices to be encapsulated, this is, according to our knowledge, the first study that experimentally showcases the remanufacturing of PSCs with a module-like architecture (i.e., including back-glass, encapsulant, and an edge-seal). We estimate that the GWP of PSM production can be reduced by 24%, or even 33% when the remanufactured modules show no PCE reduction compared to virgin modules through the described remanufacturing method while also reducing its overall environmental impact. Further, we show that the CO_2_-footprint of electricity generated by (remanufactured) PSM systems can become lower than that of c-Si, even at comparatively low lifetime and PCE. Correspondingly, we highlight that a substantial reduction in environmental impact can still be achieved by enhancing device PCE and stability. Overall, this work uniquely combines analytical and experimental methods to assess the sustainability of an emerging perovskite PV technology and to develop efficacious methods to improve it further toward a more environmentally conscious energy generation system.

## Experimental Methods

### Materials

Fluorine-doped tin oxide glass substrates TCO22-7/LI (sheet resistance 7 Ω/sq), silver paste Elcosil SG/SP, titania paste Ti-Nanoxide T165/SP, zirconia paste Zr-Nanoxide ZT/SP, carbon-graphite paste Elcocarb B/SP, and methylammonium lead iodide perovskite solution with 5-ammonium valeric acid additive (5-AVAI) were provided by Solaronix SA. Acetone was purchased from Carl-Roth, and ethanol was purchased from Alcosuisse. Titanium diisopropoxide bis(acetylacetonate) (75% in isopropanol), Hellmanex, and isopropanol were purchased from Sigma-Aldrich. Thermoplastic polyolefin (TPO) ENLIGHT XUS62250 was obtained from FirstPVM, and polyisobutylene (PIB)-based edge seal Solargain Edge Tape was obtained from Quanex.

### Fabrication of Perovskite Solar Cells with Carbon-Based Electrodes

Devices were fabricated on 10 × 10 cm^2^ plates of FTO-coated glass. First, a laser pattern defined the cathode and anode areas with an automated fiber laser. After that, the substrate was subjected to sequential cleaning steps in 1% aqueous solution of Hellmanex, acetone, and isopropanol, respectively, (15 min each) in an ultrasonic bath and subsequently dried in air. The thin compact titania layer (c-TiO_2_) was grown by spray-pyrolysis on a hot-plate set to 450 °C, using a glass mask to protect the contact areas. A volume of 20 mL of titanium diisopropoxide bis(acetylacetonate) diluted in absolute ethanol (1:160) was sprayed with oxygen as a carrier gas, and warming was prolonged for 30 min before allowing the sample to cool down. For the manufacturing of CPSCs, an array of 4 electrodes was subsequently defined by screen-printing silver contacts, m-TiO_2_, ZrO_2_ and carbon paste using a 100–40, 165–30, 90–48, and 43–80 mesh stencil, respectively (the number of strands is per cm). After printing the wet film, each screen-printed layer was allowed to dwell for 10 min before drying at 120 °C for 10 min, followed by a firing step at 500 °C (or 400 °C for carbon) for 30 min, after a 30 min ramp.

Then, a perovskite precursor solution was deposited selectively on the area of interest by inkjet with a 10 pL droplet volume and a spatial resolution tuned to match the desired quantity. The optimal resolution was determined to be 1200 × 1200 dpi. Same processes were used to manufacture perovskite solar modules with carbon-based electrodes with 12 series-interconnected cells except for the aperture area of the screen-printing mesh and perovskite filling procedure. For P1, laser ablation was used, while P2 and P3 were formed by gaps in the screen apertures during the screen-printing the porous layers. Due to high number of strands per inch (see above), suitable paste rheology and highly accurate alignment of the substrate and screen via custom-made camera alignment setup, the dead area (between P1 and P3) < 500 μm could be achieved, resulting in a high gFF of >93%.

The wet samples (cells and modules) were then moved to an oven set to 50 °C, where they were dried for 10 min, thus forming the perovskite crystals in the porous electrode structure. The resulting devices were submitted to heat and damp treatment at 40 °C and 75% RH for 150 h. according to the previously reported method by Hashmi et al.^49^ After the damp treatment, the devices were encapsulated with TPO and PIB by a home-built vacuum laminator for 10 min at 110 °C.

### Characterization

The current-density and voltage curves of solar cells were measured with a source meter at a scan rate of 5 mV/s using a class A solar simulator providing 100 mW/cm^2^, simulated AM 1.5G illumination, and corrected for spectral mismatch. The same equipment was used for obtaining *IV* curves of the modules, but the scan rate was set to 100 mV/s in this case. SEM/EDX images were obtained using Zeiss EVO 10 scanning electron microscope.

### Life-Cycle Assessment

The details of the life-cycle assessment (e.g., scope definition, indicators, assumptions) can be found in the Supporting Information.

## Supporting Information Available

Additional experimental details and analysis (PDF)
(XLSX)
