# Supplementary material for: Remanufacturing Perovskite Solar Cells and Modules–A Holistic Case Study
Source: ACS Sustain Resour Manag. 2024 Jan 31;1(3):417–26. doi: 10.1021/acssusresmgt.3c00042 (PMC10983827; doi:10.1021/acssusresmgt.3c00042)
Supplement: Supplementary file 1 — rm3c00042_si_001.pdf [file rm3c00042_si_001.pdf]

Remanufacturing Perovskite Solar Cells and Modules – a Holistic Case Study

Dmitry Bogachuk*†, Peter van der Windt†, Lukas Wagner, David Martineau, Stephanie Narbey, Anand Verma, Jaekeun Lim, Salma Zouhair, Markus Kohlstädt, Andreas Hinsch, Samuel D. Stranks, Uli Würfel, Stefan Glunz*

† These authors contributed equally to this work

*Corresponding authors

Supporting Information

Supplementary Note 1

Due to the early stages of technological readiness of PSCs, there is no scientific consensus on how (encapsulated) PSMs should look like. The aim of the PV module encapsulation is to impede moisture and oxygen ingress, provide high light transmission (when it is at the front side), a good mechanical adhesion to the substrate and a high resistance to degradation. Ideally, the encapsulation should also allow for easy recycling. Even though polymer-film encapsulation has shown promising results in keeping water and moisture out of PSCs, the technique is largely unproven on larger scales, so only encapsulation with a glass back-sheet is considered in our study.[1] Although ethylene-vinyl acetate (EVA) is traditionally used as encapsulant in PV modules, a byproduct of EVA decomposition is acetic acid, which degrades the perovskite absorber layer.[2–4] The most notable alternatives, with good moisture protection, are ionomers and thermoplastic olefins (TPO), of which the latter offer better adhesion and superior transmittance.[3,5] Additionally, contrary to EVA which forms adhesion via cross-linking bonds, TPOs contain thermo-reversible bonds between the encapsulant and glass which can be broken upon thermal treatment. In theory, modules equipped with TPO encapsulants should therefore also be easier to remanufacture than modules with EVA.[5] In addition to the TPO, an edge seal is required to retard moisture ingress from the sides, for which a polyisobutylene (PIB)-based edge seal with desiccant is used in this study. Alternative edge seals exist, but PIB-based edge seals have been traditionally used in other PV technologies and provide superior moisture and oxygen ingress protection.[6–9]

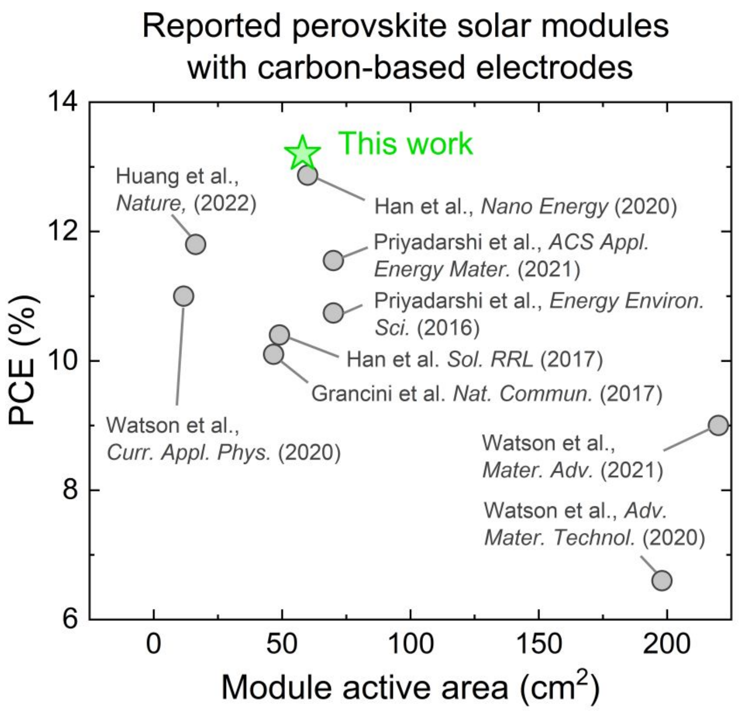

Figure S 1: Power conversion efficiencies (PCEs) of perovskite solar modules with carbon-based electrodes having different active areas in comparison to this work

Supplementary Note 2

Life Cycle Assessment

Life Cycle Assessment

In order to approximate the environmental impacts of (future) commercialized perovskite solar module (PSM) production, recycling and remanufacturing, a prospective ex-ante (predictive), cradle-to-grave life cycle assessment (LCA) is conducted in this paper. Specifically, an attributional LCA is performed, which is used to find the environmental impact of a product and identify environmental hotspots in its life cycle. The LCA is conducted using the SimaPro software, and the majority of Life Cycle Inventories (LCI) are taken from the Ecoinvent 3.7 LCI database.

1.1. Life Cycle Assessment Guidelines

The LCA methodology is largely governed by the international Organization for Standardization (ISO) 14040 &14044 guidelines, that provide standardisation for LCAs regarding general principles and requirements.[10,11] These guidelines were followed in this research, as well as LCA guidelines provided by the IEA PVPS for PV specifically, that were developed to provide consistency for the comparison of LCA results between different PV systems.[12] The ISO guidelines describe four stages that are generally used in an LCA:

1. Goal and scope definition
2. Inventory analysis
3. Impact assessment
4. Interpretation

The goal and scope definition are briefly discussed below. Additionally, ex-ante LCAs require an extra step for assumptions about the scaling up of the technology to allow for a more accurate comparison to mature reference technologies. [13,14]

1.2. Goal definition

The aims of this LCA are to identify environmental hotspots of PSMs produced on a large scale and compare the total environmental impact per impact category to reference PV technologies to assess the environmental viability of industrial PSM production. Additionally, this research aims to assess the identified PSM recycling strategy and explore whether this could potentially reduce the environmental impacts associated with PSM production.

Due to the explorative nature of this LCA, a baseline production (e.g. materials and steps used to create a PSM) and recycling process are first identified. These are conceptually scaled up to approximate the energy and material requirements of these processes if they were to be acted out on an industrial scale. This is necessary because these processes are currently only conducted on relatively small scales (10x10 cm^2 modules). Such small, lab-scale processes are inherently inefficient and may not accurately predict the quantities of material and energy used in actual (industrial, large scale) production processes, as sufficient waste streams for viable recycling and remanufacturing practices can only be achieved upon industrial production. Further, PSM LCAs based on lab-scale production are often based on the linear extrapolation of energy and material flows of lab-scale processes (e.g. spin coating), which are not viable for industrial production and have high energy and material losses. [15,16]

The PSM production (lab-scale) baseline used in this LCA is the process as described above (as done at Solaronix SA) and the recycling baseline is the process described in the main body of this paper.

Based on this baseline, we constructed a conceptual model of (future) industrial PSM production and recycling. To estimate how the material consumption of these processes if performed on an industrial scale, we consulted frameworks on how material use scales with production. The estimates on energy use during production and recycling were mostly gathered from energy consumption data of machinery used in existing (industrial) PV systems (e.g. CdTe and c-Si). These estimated material and energy flows were finally used as inputs to the LCA shown in this paper.

Correspondingly, the presented figures on environmental impact of PSMs rely on some assumptions and should be interpreted with caution. The assumptions for the conceptual model and the LCA are further explained in section 1.6.

1.3. Scope definition

The life cycle assessment conducted in this paper follows the LCA guidelines provided by the IEA PVPS.[17] It is a 3rd order LCA, where all processing and transport during the life cycle (from raw material extraction to EoL) is considered, including capital equipment and infrastructure.[18] A schematic of the reference system and its boundaries can be seen in the Fig. S2.

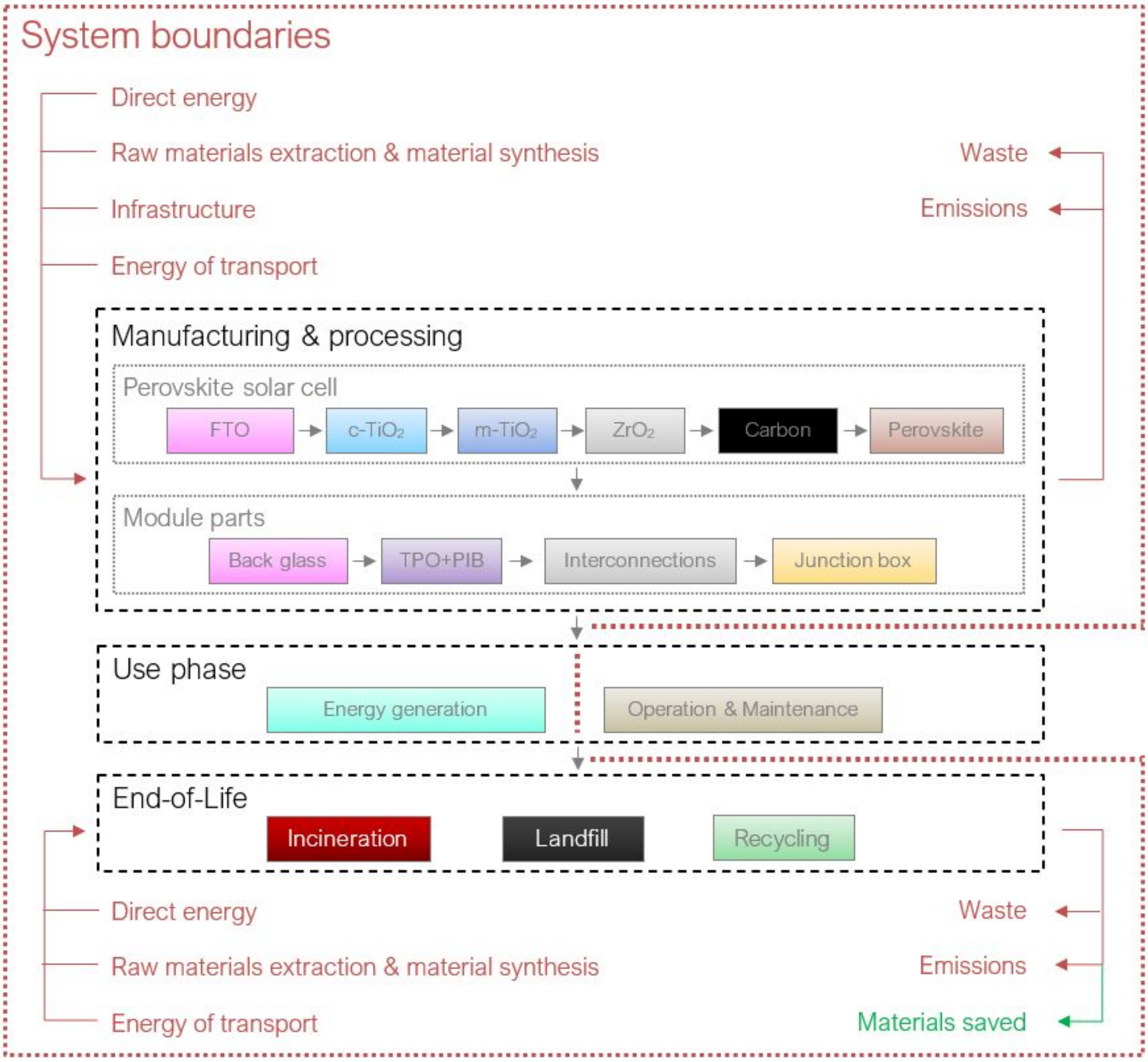

Figure S 2: Defined boundaries of system considered in the life cycle assessment (LCA)

For the capital goods used in the production of PSMs in this study, data from other technologies (e.g. inventory data for a c-Si plant) are used, as no data exist for PSM production. This means that data on the production of specific machinery (these are not the same for different PV systems) may not be entirely accurate, but such inputs are generally found to be negligible in PV LCAs.[19,20] Transport is considered here as it can contribute significantly to the overall environmental impacts of a system. It may be especially important to differentiate between EoL scenarios, as recycling or remanufacturing facilities are usually less abundant than incineration or landfill locations, and the extra distance travelled is often found to be one of the critical parameters for the environmental viability of recycling.[21,22]

For the use phase, only the generated energy (adjusted for solar irradiance when compared) is considered, as generally no significant impacts from operation and maintenance are observed. [21,23–25] For PSMs specifically, there are concerns of (toxic) lead iodide (PbI_2) leakage during

the use phase, but possible impacts have been found to not show up in LCAs, even when it is assumed that all PbI_2 leaks into the soil (or water) during the lifetime.[26] Balance of System (BoS), e.g. inverters, cabling and mounting (in this work: slanted-mount) systems are also included in this study, as they often have a significant contribution to the overall environmental impacts of PV systems.[17] To stress the effects of PSM recycling, however, the environmental impact comparison of PSMs with and without recycling will also be shown without the impacts of BoS. EoL of BoS parts were not considered, as these are removed from the module before EoL treatment of PV modules and have separate waste streams[27] that are considered outside of scope for PSM EoL.

1.4. Functional unit

As the future lifetime of PSMs (and how recycling would affect this) is currently uncertain, the results here are mainly reported per kilowatt peak (kWp) in the main text. Still, for comparison with other technologies, 1 kWh should ideally be used as the functional unit.[17] Correspondingly, the impacts are also reported per production of 1 kWh of alternating current (AC) electricity with different estimates for the lifetimes and efficiencies of recycled, remanufactured and virgin devices.

1.5. Indicators

The environmental profile typically includes a number of different indicators, namely:

- Global warming potential (GWP)
- Ozone depletion potential (ODP)
- Ionizing radiation potential (IRP)
- Ozone formation potential (OFP)
- Fine particulate matter formation potential (PMFP)
- Terrestrial acidification potential (TAP)
- Freshwater eutrophication potential (FEP)
- Marine eutrophication potential (MEP)
- Terrestrial ecotoxicity potential (TEP)
- Marine ecotoxicity potential (MEP)
- Abiotic depletion potential (ADP)
- Abiotic depletion potential – fossil fuels (ADPF)

- Human toxicity potential, cancer (HTPc)
- Human toxicity potential, non-cancer (HTPnc)
- Freshwater ecotoxicity potential (FETP)

1.6. Assumptions

Location and year

The (theoretical) location of this study is Fraunhofer ISE in Freiburg im Breisgau (Germany). Thus, the production of PSMs is assumed to take place in Freiburg im Breisgau. Correspondingly, the background processes (e.g. electricity mix and chemicals produced) are modelled using regional, e.g. Germany or Europe (RER) LCI data, unless the production site is known to be somewhere else. When not available, global market processes were used. Additionally, the solar irradiance of Freiburg im Breisgau (1429.2 $kWh/m^2/y$, based on an optimal tilt[28]) was used to calculate the energy yield. The background data used are from the year 2021 (or as recent as possible), as determining the environmental impacts of PSM production with current background processes (instead of a future electricity mix) allows for more accurate comparison to reference PV technologies.

System performance

The PCE used for virgin (non-remanufactured) CPSMs produced by Solaronix SA in this study is 13.2%, as obtained from the JV-measurements (Fig. S14). As the production system is based on a conceptual model of industrial PSM production, the lifetime should reflect that of a product viable for market introduction. Here, we estimate a lifetime of 15 years for the reference module, which is thought to be a lifetime at which PSM power generation can be cost-competitive with other PV technologies.[29–31] The PSM is considered to be monolithically interconnected (through laser scribing) with an active area or Geometric Fill Factor (GFF) of 88.7% (leading to a total module efficiency of 11.71%). Although the CPSM manufactured in this work (Fig. S3) have a GFF of 93.4%, we expect this value to be lower with an increase in device area. Therefore the GFF used in the LCA is based on the assumption that industrially produced modules will have a cell width of 4.9 mm (for n-i-p architectures with 10 Ω sheet resistance), which is found to achieve a good balance between geometrical losses (dead area) and resistive losses (series resistance goes up when the cell area is increased).[32–35] Additionally, a P2 scribe

is with a width of 150 μm is considered, which should give enough safety margin to avoid too narrow scribes (leading to increased series resistance) and avoid high series resistances due to incomplete scribing.[36] Resistive losses from the P1 and P3 scribe should already be negligible for widths of 10 μm,[36] but a conservative estimate of 25 μm is used here, with safe areas (areas introduced between the p1, p2 and p3 scribes to reduce the risk of misalignment) of 50 μm each, giving a total dead area width of 300 μm. Including an edge seal width of 1.2 cm on each side (this provides moisture protection compatible with 25-year module lifetimes[37]) and a module size of 1.2x0.6m, this gives a GFF of 88.7%.

As PSMs are not commercially produced yet, Degradation Rate (DR) and Performance Ratio (PR) were obtained from other PV systems and background data. The PR is measured as the ratio of average AC output compared to the rated output of a solar panel and is comprised of many different losses. In this research, a PR of 0.846 is assumed, with the individual losses (and assumptions) from Table S1. The DR is assumed to be such that the efficiency of the PSMs assessed here decline linearly to 80% of their rated power (DC, not including losses) at the end of its lifetime, which is the typical guarantee offered by manufacturers of commercial PV systems.[38,39] It is sometimes found that degradation does not happen linearly[40,41] and more empirical research would be needed for PSM systems specifically. Still, an approximately linear decline in efficiency is deemed an accurate predictor,[42] because most of the degradation tends to come from short circuit current (J_{sc}) losses from encapsulant discoloration, which follows a linear pattern.[43] Over the whole lifetime (including DR), the PR of the reviewed system here is then 0.7614. This is also assumed for the comparison with CIGS. For c-Si modules, a lifetime PR of 0.85 is assumed, based on its warranty.[25] The comparison with c-Si in this report entails c-Si Passivated Emitter and Rear Cells (PERC) specifically, as this technology makes up the bulk of c-Si market share[44].

Table S1. Parameters and contribution of the different losses to the total loss and PR.

		**Source**	**Comment**
Soiling loss	4%	45	Average on rooftops in Germany
Inverter loss	2%	39,46	
Mismatch and Dc cabling loss	0.90%	39	
Shading loss from other panels	0%	47	Typically, no panel shading losses on rooftops.
AC and transformer loss	2.20%	39	

Availability loss (maintenance etc.)	1.00%	[39]	
Temperature loss	5%	[48,49]	Based on an average temperature coefficient of a-si, CIGS and CdTe thin film and a nominal operating temperature of 45 ^{o}C (STC is 25 ^{0}C)
**Total**	15.40%		PR 0.846

The lifetimes of BoS parts are based on [38], and are considered the same for all PV technologies mentioned in this report (Perovskite and c-Si), irrespective of lifetime of the module. They are 15, 30 and 30 years for inverters, slanted mount (rooftop) installation and cabling, respectively. In order to provide a fair comparison between technologies, the background parameters (e.g. irradiation, BOS lifetimes) of the c-Si impacts shown in this paper have all been corrected to match the parameters of the PSM LCA presented here.

Based on the processes considered within the scope of LCA, the environmental profile of the CPSMs has been constructed in Fig. S3.

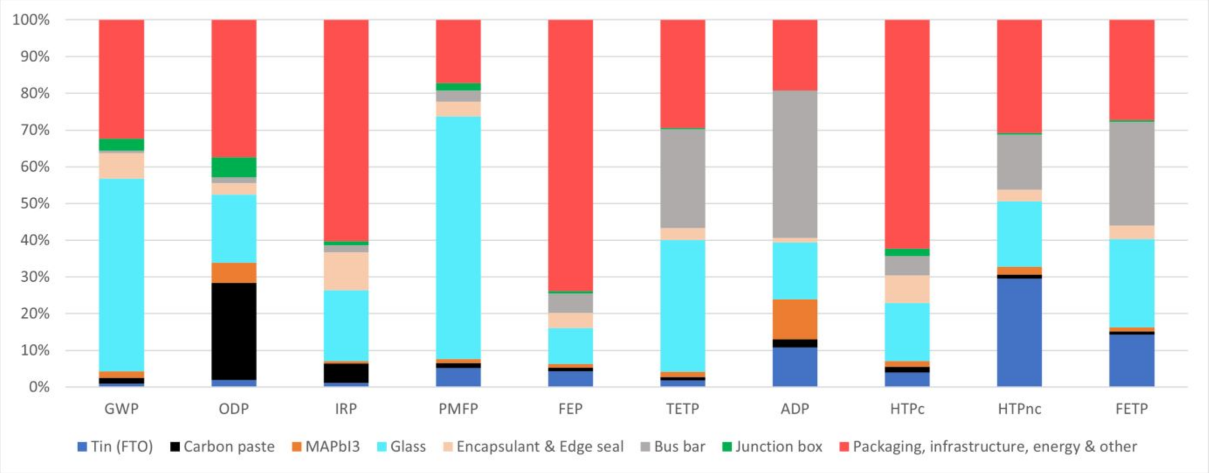

Figure S 3: Environmental profile of CPSMs, showing the contribution of each component to each environmental indicator, discussed earlier.

As mentioned in the main manuscript, here we consider the GWP of the module per kW_p as the main environmental indicator. The environmental profile was assessed based on the data from IPCC 2011, gwp 100a, V1.03 databases.

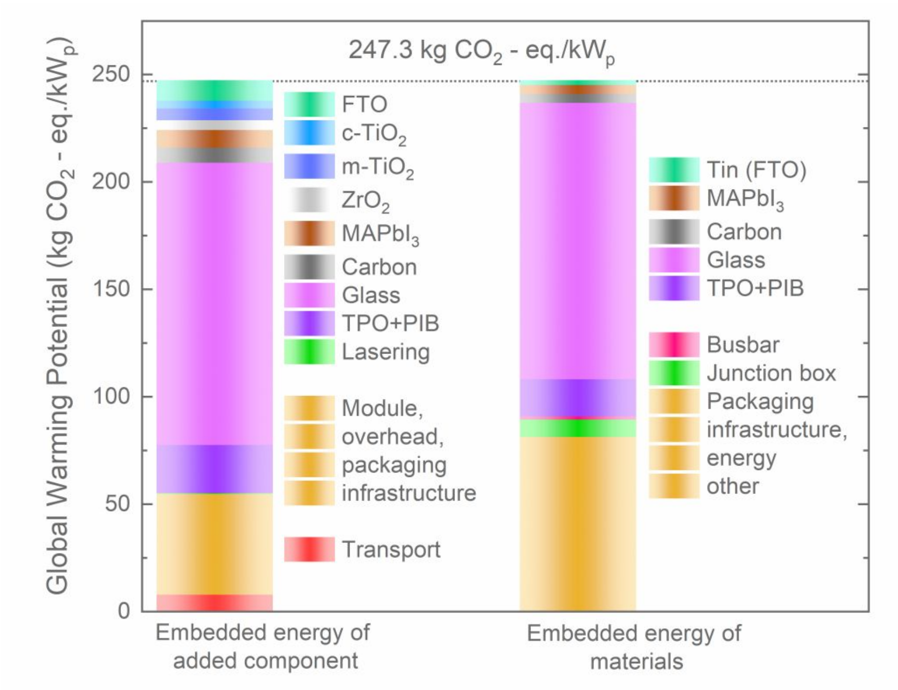

Figure S 4: Distribution of component contribution to the total GWP of a PV module

Fig. S4 demonstrates the distribution of individual contributions of embedded energy of each added module component and materials in the total GWP of the CPSM. Yielding complementary information to Fig. 1c of the main manuscript.

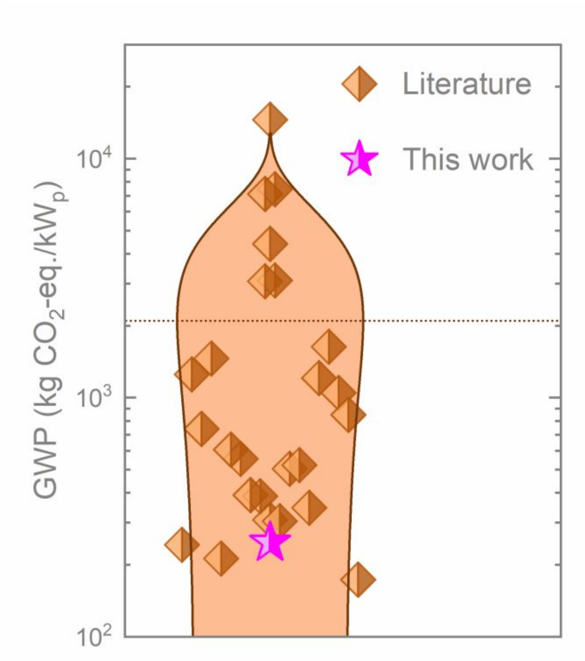

Figure S 5: Calculated GWP based on LCA reports of perovskite PV modules in literature[50] in comparison to this work

Energy and Material Assumptions

As mentioned, the LCIs of background processes were mostly obtained from Ecoinvent v3.7. When no specific data on production processes or transport was available, the market activities were selected, which comprise the consumption mix of a certain product or process and include average transport distances. Electricity used for small-scale production processes are assumed to be low voltage, while medium voltage is assumed for larger scale chemical industry and for industrial PSM manufacturing.

Chemicals

For many chemicals, no readily available LCI data exists. These LCIs were constructed with the help of patents and literature review. However, these often describe processes on a very small scale, which typically deviates from how chemicals are industrially produced. As such, small scale syntheses tend to be energy and cost inefficient.[51–53] Accordingly, the production of these chemicals were theoretically scaled up using assumptions provided by Piccinno et al.[52] The most important calculations from this framework are discussed below.

Regarding materials, a 20% decrease in solvent use (when there are no fundamental limitations to using less solvent) are assumed when only data on lab-scale production is available. To estimate the energy consumption per kg of chemicals produced, a series of equations is used. Typically, for these equations, different parameters (such as diameter and thermal conductivity of reactors) are required. Estimates of these parameters are provided in [52], and depend on the size of the reactor. For processes with no specific data availability on such parameters, a 1000 L reactor is assumed in this study based on their recommendations.

The energy to heat reactions is calculated based on the energy to reach the reaction temperature (Q_{heat}), energy to compensate for eventual heat losses (Q_{loss}), and the heating efficiency (η_{heat}). Q_{heat} is calculated as follows:

$$Q_{heat} = C_p * m_{mix} * (T_r - T_0) \#(1)$$

with C_p as the specific heat capacity (J/kg*K), m_{mix} the mass of the mixture, and T_r and T_0 as the reaction temperature and the ambient temperature (20 ^{o}C) respectively. For Q_{loss} the following equation is used:

$$Q_{loss} = A * \frac{k_a}{s} * (T_r - T_{out}) * t \#(2)$$

with A as the surface area of the reactor, k_a the thermal conductivity of the insulation material, s the thickness of insulation and t the reaction time. All these data are dependent on the size of the reactor used for the reaction, but unless otherwise mentioned, a 1000 L reactor is used with A 5.899 m^2, k_a 0.042 W/m*K and s 0.075 m (rate of heat loss per kelvin of 3.303 mW/K/L) . The default η_{heat} is 75%, and the formula to calculate the total heating energy is then:

$$Q_{react} = \frac{Q_{heat} + Q_{loss}}{\eta_{heat}} \#(3)$$

Additionally, if certain solvents or chemicals are known to evaporate in the reaction, the enthalpy of evaporation (ΔH_{vap}) is also added (divided by η_{heat}).

Drying is typically used to vaporise the remainder of wet fraction that has not been removed in the previous steps and is often necessary to purify products. It is similar to the heating step, but often takes place in an oven instead of a reactor. The energy needed is calculated as follows:

$$Q_{dry} = \frac{C_{p,liq} * m_{liq} * (T_{boil} - T_0) + \Delta H_{vap} * m_{vap}}{\eta_{dry}} \#(4)$$

with C, m and ΔH_{vap} denoting the specific heat capacity, mass and the enthalpy of evaporation of the liquids to be evaporated respectively. η_{dry} can vary a lot but is assumed to be 80% (including heat recovery) in the chemical industry.

The energy for distillation is difficult to estimate, and may vary a lot depending on the specific reaction and solvents used,[53] so for distillation steps a default 1.53 kg of steam per kg is assumed, based on an average of empirical values.[54]

Many reactions are stirred (or homogenised) as well. To estimate the stirring energy, the following equation is used: $E_{stir}(J) = \frac{N_p * \rho_{mix} * N^3 * d^5 * t}{\eta_{stir}} \#(5)$

with N_p the type of stirrer (dimensionless, specific to the type of impeller), ρ_{mix} the density to the reaction mixture (often estimated), N the rotational velocity of stirring and d the diameter. In the default (1000L) reaction, N_p, N and d are assumed to be 0.79 (axial flow), 1.1417 L/s and 0.373 m respectively.

For filtration and centrifugation, it is mentioned that the energy use can vary widely (from 1 to 10 Wh per kg of dry material).[52] As these energy requirements are sufficiently low and often insignificant compared to the heating energy, the upper value of 10 Wh per dry material is used in this study.

Lastly, reaction mixtures are often transferred (pumped) between different reactors, which is typically done through pipes. Piccinno et al. provide calculations to calculate the exact energy requirements for this,[52] but these are deemed too extensive given the lack of exact data on pumping and the small contribution of pumping energy to the total. As such, a default energy of 55 J/kg is used for pumping, assuming that all liquid mass is transferred once throughout its reaction (if no specific data is known) based on.[55]

Vacuum drying is not included in the framework presented by Piccinno et al.,[52] as the energy is typically difficult to quantify and case specific.[56] Here, the power use from vacuum drying is approximated using the following equations (based on rotary piston vacuum pumps, which is often the vacuum pump of choice in the chemical industry:[57]

$$P\ (kW) = 4.242 * SF^{1.088} \#(6)$$

where SF denotes the size factor of the vacuum pump, calculated as follows:

$$P\ (kW) = \frac{2.2 * \dot{m}}{p} \#(7)$$

with $\dot{m}$ as the mass flow rate (kg/h) of the gas to be evacuated and p as the operating pressure (mmHg).[55,58] A standard leak rate (air back into the vacuum chamber) of 10^{-6} Pa/m^3/s is assumed.[59] This calculation only holds for low vacuum (>1 mbar), however. For higher vacuum, the energy requirements are based on the nominal power of a middle-sized vacuum

pump.[59] Cooling and freezing are also not included in the framework by Piccinno et al.[52] Cooling and freezing energy are therefore calculated based on the temperature difference needed and the specific heat capacity (J/kg/K) of the reaction mix, with a Coefficient Of Performance (COP) of 1.43 and 2.28 for cooling and freezing respectively.[60]

Typically, reaction mixtures are filtered before a final (vacuum) drying step. The leftover moisture content (to calculate the drying energy with) in these reaction mixtures is assumed to be 25%, based on data of an industrial chemical filter press.

When no information on the synthesis of specific chemicals was available, the average energy use per kg product of a big chemical production site (Gendorf) are used based on[61] (2.76 MJ/kg, 2.06 MJ/kg and 0.24 MJ/kg for natural gas, electricity and steam respectively).[62]

Reaction yields were obtained from literature where possible, and assumed to be 95% when no data is available, based on Ecoinvent standards.[63] Fugitive emissions (release of gases during reactions) are difficult to quantify and their amount may vary, but are typically low.[64–66] As a conservative estimate (when no process data is available), 0.2% fugitive emissions are assumed for any chemical that is heated above its evaporation temperature, while the remainder of solid and liquid waste is assumed to go to water, based on [61,67]. Hazardous substances (according to the list by the Environmental Protection Agency, EPA[68] or substances that may be contaminated are assumed to go to hazardous waste incineration. Gaseous by-products are usually scrubbed off (and sometimes recovered) in chemical reactions,[69–71] so these were modelled as such (taking into account the extra inputs for scrubbing).

Solvents used in the production of chemicals are also considered to be incinerated, as no data specific product data is available and the energy requirements for solvent recovery are product specific and can vary widely.[52,53] As the materials used (and the faith of solvents) in PSC production are not estimated to have very large relative environmental impacts based on previous LCAs[72–74], hazardous waste incineration is used as a conservative estimate.[52]

Lastly, specialty chemicals (e.g. PbI_2 & MAI) are assumed to be produced in relatively large quantities (at least per kg, as opposed to per gram or less, which is sometimes assumed, e.g. in [75]) and then stored, as this is generally done and more economical for chemical manufacturers.[51,76,77] Specifically, these special chemicals are considered to be produced using the parameters associated with the smallest reactors in the processes described in the framework by Piccinno et al.[52] Compared to the default assumptions (1000L reactor), this entails a higher rotational velocity of stirring (N) of 3.052 L/s, higher heat loss per Kelvin of 7.12 mW/K/L and a lower η_{heat} of 72%. Further assumptions and calculations are described per (type of) precursor below. Additional, specific assumptions can be found in the LCIs per product in the Supplementary Excel File**.**

Pb- based and non-metallic cation precursors

The LCIs for Pb-based precursors (PbI2, PbCl2, PbBr2) in previous literature were found to often not include extra purification steps.[74,75,78] These studies likely underestimate the impacts of Pb-based precursors, as the purification step (vacuum drying) is found to be necessary for good device performance. Wakamiya et al.[79] found that even 0.1% water content in PbI_2 samples already reduced the efficiency of their devices from 10% to 5.23%, while other papers have also reported the necessity of Pb_x purification to produce efficient PSCs (e.g.[80–83]).

The identified Pb_x synthesis routes used in other researches often use a similar principle and are based on precipitation reactions[80–82,84,85], which is likely also used industrially (at least by Sigma-Aldrich to produce $PbCl_2$)[86]. The general principle for these reactions is that two solutions of soluble salts (with water) are mixed and heated (to roughly 100 ^oC) to make the product more soluble, after which the solution is cooled down and the desired product precipitates (due to its low solubility). The Pb_x needs to fully dissolve during this reaction, in order to induce recrystallization and produce good quality Pb_x crystals.[80] It is not known which exact precursors chemical manufacturers use to make PbI2, PbCl2 and PbBr2, but the high energy requirements of heating water for these reactions (and subsequent purification) are found to dominate the environmental impacts. Thus, the LCIs of PbI2, PbCl2, and PbBr2 presented here likely serve as a good approximation, even if the precursors used by chemical manufacturers are different.

For the purification of the PbI2, PbCl2 and PbBr2, a high vacuum is needed of around 0.27 mbar[79,81,83]. The energy requirements are based on the nominal power of a middle-sized vacuum pump.[59]

The LCI for PbI_2 here assumes a reaction between lead nitrate and potassium iodide, as this is found to produce high quality crystals (better than when lead oxide is used) [530]. The LCI is based on the synthesis described by Schlessinger[84] (95% yield based on potassium iodide), which is also roughly used in most perovskite publications[80,87,88] and is found to deliver perovskite layers with similar quality to pre-bought PbI_2.[81]

For the non-metallic cation perovskite precursors (MAI, FAI, MABr, not including CsI), the syntheses reported in the literature generally describe more or less the same synthesis. Roughly, the precursors (a combination of either methylamine or FAAc and hydroiodic acid or hydrobromic acid, depending on the desired substance) are mixed and stirred for 2h at 0 oC, evaporated at 50 oC for 1h, dissolved in ethanol (for FAI only), then recrystallised using diethyl ether and dried at 60 oC in a vacuum oven for anywhere between 8 to 24 hours. This general procedure is e.g. reported in [89–96]. As such, this procedure (16 hours vacuum as an average of the reported numbers at 1.33 mbar), with a yield of 97%.[83], is assumed to be used in the production of these chemicals. None of the mentioned papers report how much diethyl ether is used for recrystallisation, so 5 ml per gram product is assumed for MAI, MABr and FAI, based on their similar (high) solubility[97–99] based on amounts typically used in the chemical industry.[100] For FAI, 1 ml ethanol per gram product is additionally assumed.

Carbon Paste

The carbon paste used at ISE is purchased from Solaronix SA (Switzerland). The composition of the carbon paste used is not entirely certain, as it is based on a safety datasheet of the Elcocarb B/SP carbon paste of Solaronix SA, which only lists ingredients with error margins. The carbon black to graphite ratio in the paste is also not mentioned, so a weight ratio of 1:2.6 graphite: carbon black is assumed, as this provides optimal conductivity.[101] No energy is assumed to make the carbon paste.

Solvent Incineration

Ecoinvent has no specific LCI data available for the incineration (with energy recovery) of different solvents (only one generic “spent solvent” process), even though the energy content

of solvents may vary widely. As such, individual solvent incineration processes were created for each of the solvents used in this study (for background and foreground processes). The Ecoinvent process assumes an average energy content of 21.7 MJ/kg lower heating value (LHV).[102] The individual solvent incineration processes in this study were made by altering the generic solvent using the difference between LHVs of the individual solvent and the LHV of the average solvent. 60% of this difference was then added as (extra) steam output, as in Germany waste facilities are typically located near chemical industry, where steam is supplied to manufacturers right away, with an efficiency of roughly 60%.[103] For example, for ethanol, with an LHV of 26.7 MJ/kg, this means that ((26.7/21.7)*0.6 =) 3 MJ of steam is additionally supplied.

Encapsulation Materials

Besides the chemicals, LCIs were compiled for the edge seal (PIB) and encapsulant (TPO) materials used in this study. TPO is made of a majority polyproplene (PP) or high molecular weight polyethylene (HMWPE) that are co-extruded with some additives to provide extra strength.[104–106] For PV encapsulation specifically, it is found that the TPO used is often HMWPE-based.[107–109] The exact composition of TPO encapsulation materials are uncertain, but spectroscopy showed that the amount of additives is very low, and the vast majority of the material consists of polyethylene.[110] Thus, the LCI used for TPO encapsulant in this study consists of only HMWPE.

The edge seal is mostly PIB-based, but also has a desiccant in it, that binds eventual water that ingresses in from the sides. [111] This desiccant can be a molecular or a reactive sieve, of which the latter typically provides better water protection[37,112,113]. As such, it is here assumed that the PIB-based edge seal uses a reactive sieve, specifically Calcium Oxide (CaO), based on [111] and [114]. The CaO content is assumed to be 22.5%, based on averages from [114] and [115].

The LCI for (fossil) isobutylene in [116] is used here. Polyisobutylene is created by cooling isobutylene to -100 ^{o}C and using lewis acids to initiate the reaction.[117,118]. The amount and type of lewis acids could not be found but, as they are only used as catalysts, their impacts are assumed negligible and only the energy to cool to -100 ^{o}C is considered here. PIB is not vulcanised[119,120].

Energy assumptions

Besides the material inputs, the energy flows of the manufacturing processes needed to be identified. Most of the energy inputs were obtained from previous LCAs and equipment data. The specific assumptions are mentioned in the individual LCIs constructed here. The largest energy consumption often comes from the heating steps. The power consumption here is based on the hourly power consumption of conveyor belt ovens and their throughput, mostly based on the Calipso LT 8lane ACD conveyor furnace from Meyer Burger. The consumption is only stated at 100 ^{o}C, 200 ^{o}C and 850 ^{o}C, so the power consumption is linearly interpolated for the m-TiO_2 (450 ^{o}C) heating step. This seems to be a fair assumption, given the small difference in power consumption between 100 ^{o}C (20 kW) and 850 ^{o}C (30 kW, both 14.1 long conveyor furnaces). The conveyor furnaces can be from 4.7 (heating length) to 35 m long, and different lengths (and different power consumption per m^2 processed) are here assumed (see

Supplementary Excel file, table 'Energy Consumption'), depending on the heating time required (e.g. longer furnace assumed for m-TiO_2 curing, which takes 30 minutes.

Some PSM manufacturing equipment also use compressed dry air (e.g. screen printing and annealing), which is often produced on location. The associated electricity use may vary, but here an average power consumption is assumed of 0.124 kWh per m^3 compressed air (6 bar).

Glass

The FTO coating takes place at a glass factory. Most of the energy consumption in APCVD is from heating (substrate and precursors). For FTO (in-line) the glass is still hot from the float glass process, from which waste heat is used to heat the precursors (confirmed through e-mail communication with Pilkington, T. Braun, April 16, 2021). This energy is already included in the float glass Ecoinvent process, so only the power to deliver and deposit precursors is considered for the FTO coating.

For (FTO) glass cutting, there are some cutting losses (often related to different glass size orders), that typically range between 0 and 10%. In this study, we assume 5% losses. Electricity consumption for glass cutting is estimated at 0.029 kWh (including compressed air) per kg.

Encapsulation

The encapsulation is assumed to be 800 µm thick (2x400 µm thick sheets on top of one another) as this should provide sufficient moisture and oxygen ingress protection. The PIB-based edge seal has roughly the thickness of the encapsulant plus the PSC stack (~802 µm) and a width of 1.2 cm (after lamination, during which it expands), which is shown to provide good moisture protection. The encapsulation process is quite energy intensive, as it requires heating, (low) vacuum and cooling steps. The electricity use is estimated from equipment data, where a power consumption of 93 kW is mentioned for a throughput of 46 modules of max 2.3x1.4 m (~148 m^2), which translates to 0.63 kWh/m^2. The encapsulation equipment can be customised to different module sizes. Here, a 20% increase in energy consumption is estimated as a conservative estimate of encapsulation of 0.6x1.2m modules (smaller equipment tends to be less energy efficient). Note that the throughput of 46 modules is based on polyolefin elastomer. Due to the lack of cross-linking agents in TPO, the throughput could probably be higher than that of POE (and the electricity consumption further reduced), but this throughput is assumed as a conservative estimate.

Manufacturing Stage

The energy use of the theoretical industrial manufacturing process are mostly based on machines used in current PV lines of other technologies, such as c-Si, and CdTe. Solvents evaporated during the process are modelled as emissions to air. Material utilization efficiencies (MUEs) are based on literature data.

Table S2. MUEs for the different deposition techniques used in PSM manufacturing.

Process	MUE	Comment
CVD	10%	
Spray pyrolysis	39.50%	

Screen printing	95%	Conservative, 99%
Slot-die coating	95%	Conservative, up to 99%

One glove box is considered per manufacturing line, as this is necessary to prepare and store perovskite precursor solution (and store PIB, which requires a nitrogen atmosphere). The precursor solution is assumed to use a DMF:DMSO molar ratio of 7:3, as this is roughly the ratio found to provide the best device performance for $MAPbI_3$.

Overhead and Workspace

The overhead electricity (for the industrial production line) includes the electricity for heating, ventilation (also to get rid of any process vapours), air-conditioning and lighting. The total workspace is firstly estimated, then the space needed for equipment is based on product data. Then, on each outer end of the manufacturing line, 3 m length is added, while between tools of a manufacturing line, 4m distance is assumed for handling (e.g. loading and offloading). To the calculated area, 10% (each) is added for support, logistic and administration areas, and another 15% is added for facility area. The electricity use is then estimated as 120 Wh/m^2/h and 50 Wh/m^2/h for heating, ventilation, air-conditioning, and lighting in the manufacturing area and in the support, logistics, administration, and facility areas respectively.

For the overhead electricity use, a factory of 2 lines of 90 MW production (~677000 m^2/year, 88.2 m^2/per hour, operation 7680 hours/year) is considered, based on the calculations shown in Supplementary Excel Table 'Overhead'. Most notably, the rate limiting steps were the m-TiO_2 annealing (30 minutes) and encapsulation. For the encapsulation, 2 machines are considered per line, while for the m-TiO_2 annealing, a rather long conveyor belt oven is considered (35.2 m heating length). This conveyor oven has a throughput of 3200 wafers (0.166x0.166m) per hour, translating to 88.2 m^2/h, which is considered as the (rate-limiting) throughput per line. The length of the conveyor oven could be further increased up to 50 m, but this would have diminishing results given that other processes would then start to limit the overall throughput. The overhead electricity calculation is shown in the supplementary excel file, table 'Overhead'.

Table S3. Overhead electricity use calculation.

	**Area**	**Electricity per area**	**Electricity per hour**	**Electricity, per m^2 of PSM**
	m^2	Wh/m^2/h	kWh/h	kWh/m^2
**Heating, ventilation, and air-conditioning**				
Gross manufacturing area	3838	100	384	2.18
Support, logistic, administration and facility areas	1727	30.0	51.8	0.29
**Lighting etc.**				
Gross manufacturing area	3838	30.0	115	0.65
Support, logistic, administration and facility areas	1727	20.0	34.5	0.19
**Total**	5565		436	3.31

1.7. EoL Life Cycle Assessment

The remanufacturing LCIs are made based on the remanufacturing procedure as detailed in the main body of this paper. The material and energy use of the lab-scale procedure likely do not reflect that of a large (industrial) procedure. Thus, to compile the LCIs, some assumptions were made on how this procedure would look on a larger scale. The assumptions specific for the remanufacturing and other EoL scenarios ('bulk recycling' and landfill) are briefly detailed below. The LCIs and more detailed assumptions can be found in the supplementary excel file (tab 'PSM EOL').

The clean removal of TPO and PIB was found to work best when the entire upper surface of the module front and back part (after separation) are covered with acetone. For a large scale procedure, although some acetone may evaporate, not much more acetone would likely be needed than the amount that is necessary to cover the entire surface of the module. To estimate the acetone needed to accomplish this, we assume an acetone bath the size of 1.26x0.66m (1.2x0.6m module plus 3 cm extra on each side to leave some room for imperfections during the procedure of dipping the module into the bath). This bath should be filled with acetone until it reaches the top surface of each module part (roughly 3.5m thick on each side after separation) plus 0.5 mm. Based on our experiments, we estimate that this amount (one bath) should be enough to separate the TPO and PIB and both the front and the back layer of the module.

Since only the front part of the module (with the active layers) needs to be put in the Methylamine/ethanol bath, we assume one bath to be sufficient for 2 modules. This should contain plenty of solution to dissolve the amount of PbI_2 on 2 modules (see supplementary excel file for calculations).

Note that the estimated amount of solvents may be on the conservative side, since industrial procedures could likely be optimized to have smaller margins (e.g. a bath with less extra area) and to re-use leftover solvents from the previous module (which we do not consider). These conservative estimates are used in order to not underestimate the environmental impacts of solvents needed for this remanufacturing method (and the overall impact of a remanufactured PSM).

The overhead electricity consumption is assumed to be around a third of that for the PSM production, since the remanufacturing procedure requires far less machinery and therefore a much smaller area. For the remanufacturing LCA, all solvents used are assumed to be incinerated (since they may contain hazardous substances) and the encapsulation material is assumed to end up in landfills, based on the IEA LCA approach for CdTE.[38]

For the bulk recycling case, only glass and copper are considered to be recycled. To assess the environmental impacts of glass recycling (not re-use or remanufacturing), the change in environmental impacts (relative to the production of a non-recycled PSM) are approximated through LCA data from CdTe recycling that follow the "avoided burden" approach.[38] Specifically, the energy inputs (e.g. hammermilling, shredding, separating the parts) for CdTe recycling are used to model the additional impacts that come with recycling of glass.[121] The avoided impacts from glass recycling are modeled through the materials and energy that are avoided by using recycled glass cullets instead of raw materials to create virgin devices (Table S1 in ref. [38]). For transport, 100 and 578 km by lorry is assumed as distance to the collection point (where the module would be placed during its lifetime) and distance from collection point to recycling plant respectively, based on CdTe recycling.[38]

The avoided burden approach is used here because no accurate data on the exact inputs of flat glass production could be found (which would be needed to follow the cut-off approach that is used for the remanufacturing LCAs in the rest of the paper). We assume this to be more accurate than estimating the energy and material inputs for flat glass production. The avoided burden of glass recycling in remanufactured PSMs is adjusted based on the relative mass fraction of glass in CdTe modules and PSMs (93.2% for PSM, 96.3% for CdTe[38]).

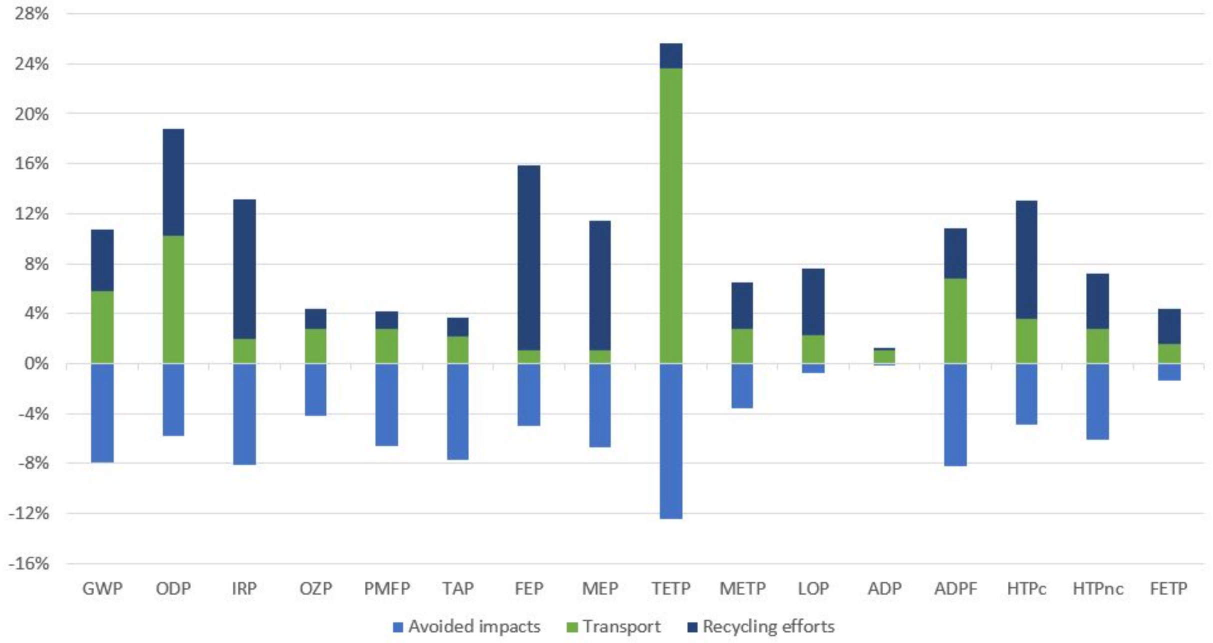

Figure S6: Complete environmental impacts related to glass recycling, relative to the environmental impacts of the production of a PSM (0%).

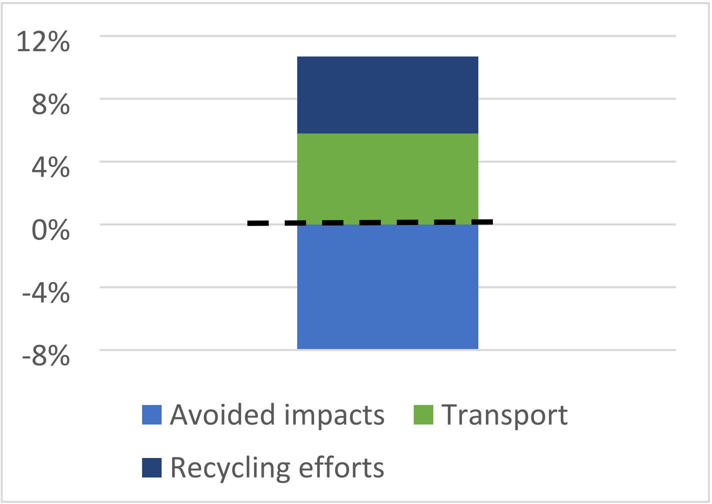

Figure S 7: GWP related to "bulk" recycling, relative to the GWP of the production of a PSM (0%). The dashed line indicates the net impact.

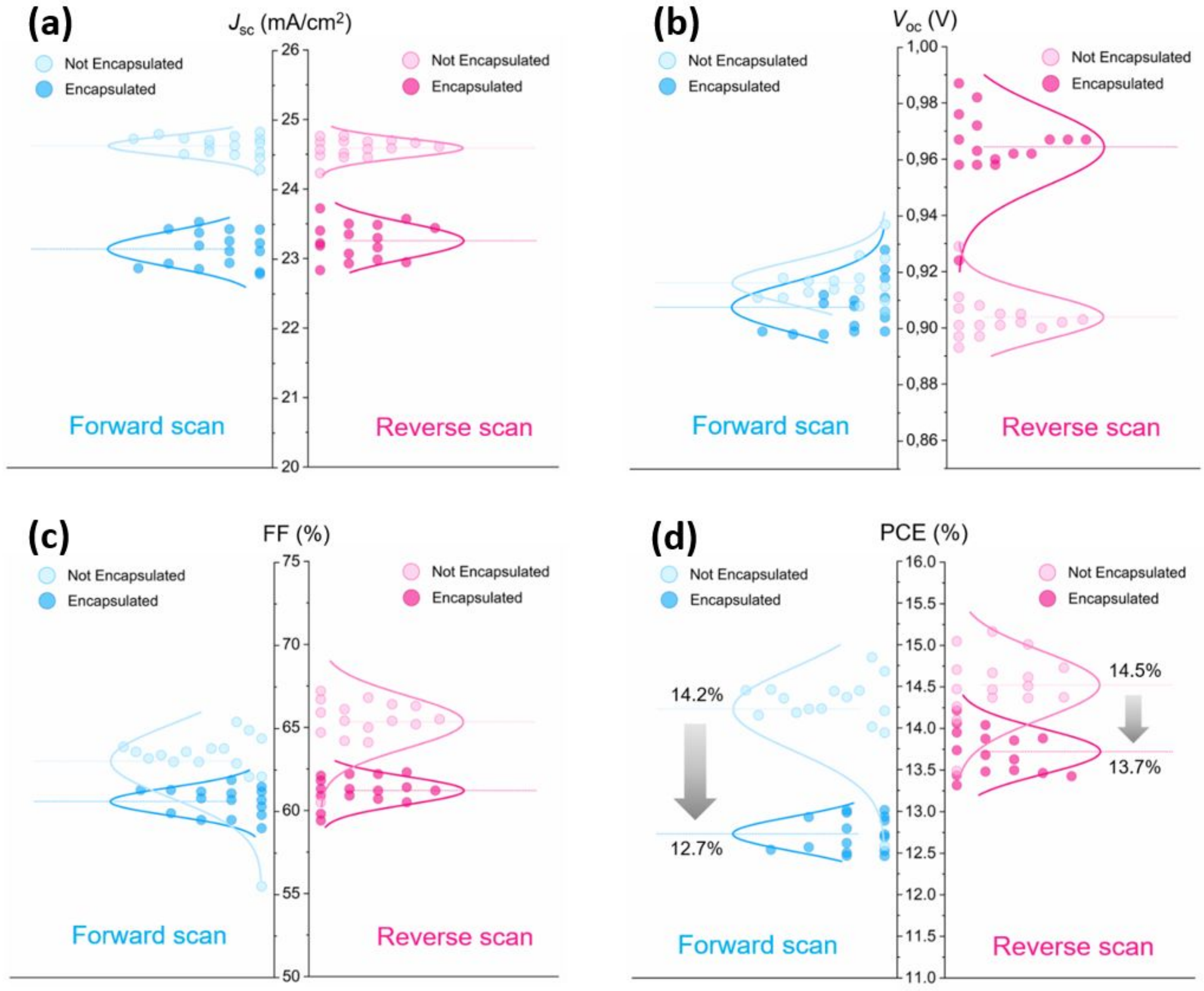

Figure S 8: Short-circuit current density (Jsc), Open-circuit voltage (Voc), fill factor (FF) and power conversion efficiency (PCE) of manufactured devices before and after encapsulation (i.e. same devices), obtained from forward and reverse J-V sweeps.

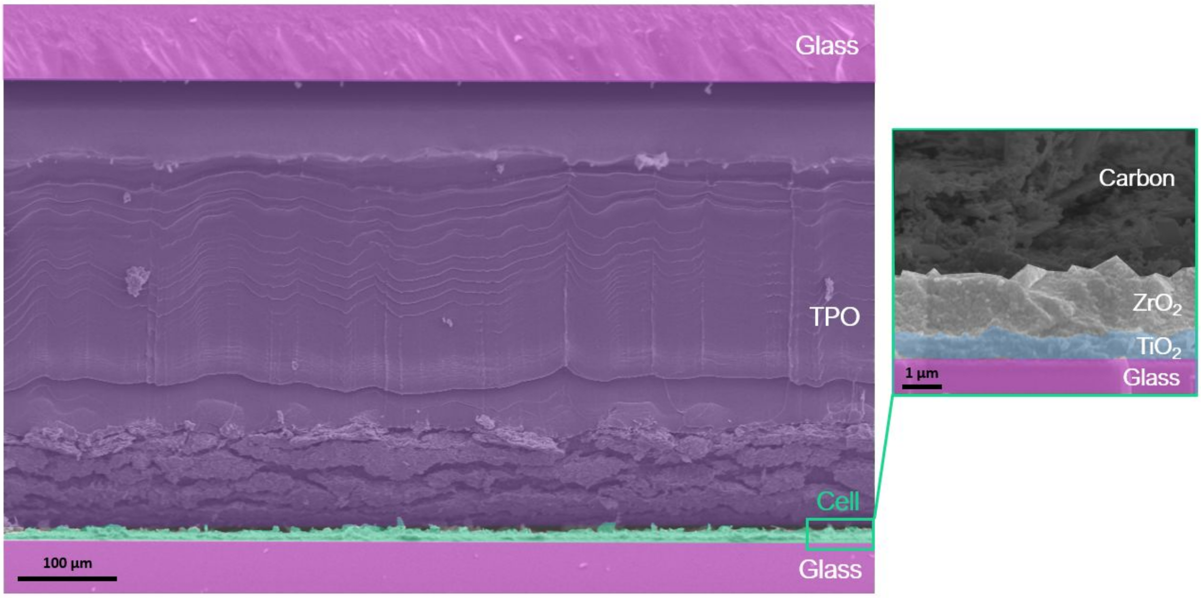

Figure S 9: Cross-sectional scanning electron microscope image (SEM) showing the structure of an encapsulated perovskite solar cell with carbon-based electrode. The color scheme also corresponds to the legend in Fig. S2 for enabling a clear link between a cell component and its environmental footprint.

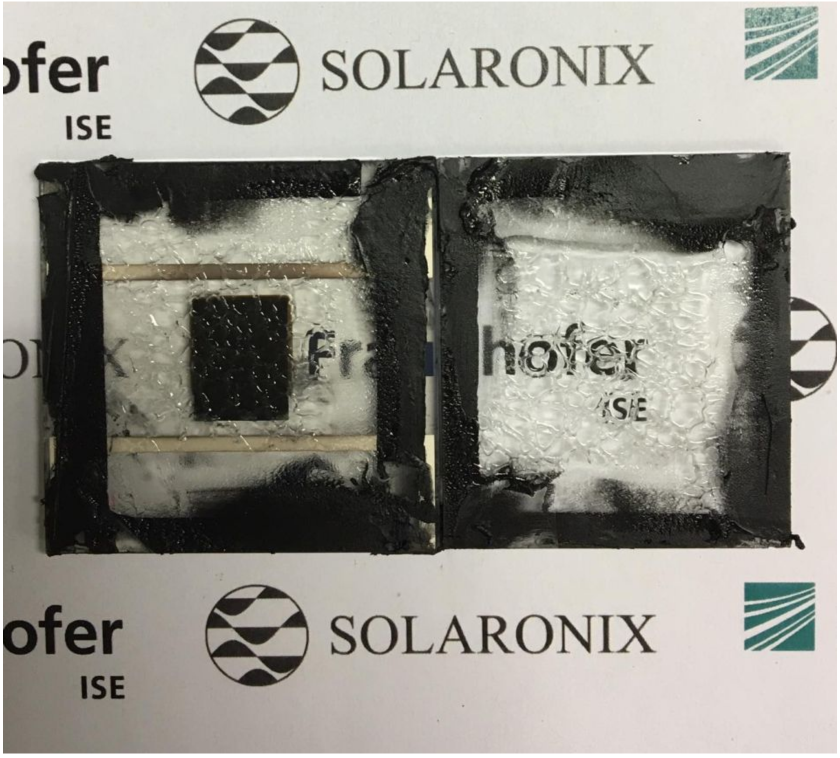

Figure S 10: Cell and back-glass with PIB and TPO after thermal-assisted separation

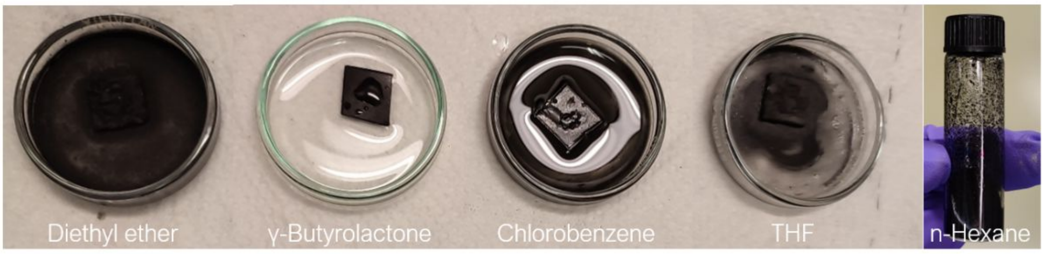

Figure S 11: Dissolution of PIB in screened solvents, none of which however could fully dissolve PIB, although we note that n-hexane exhibits the strongest dissolving capability

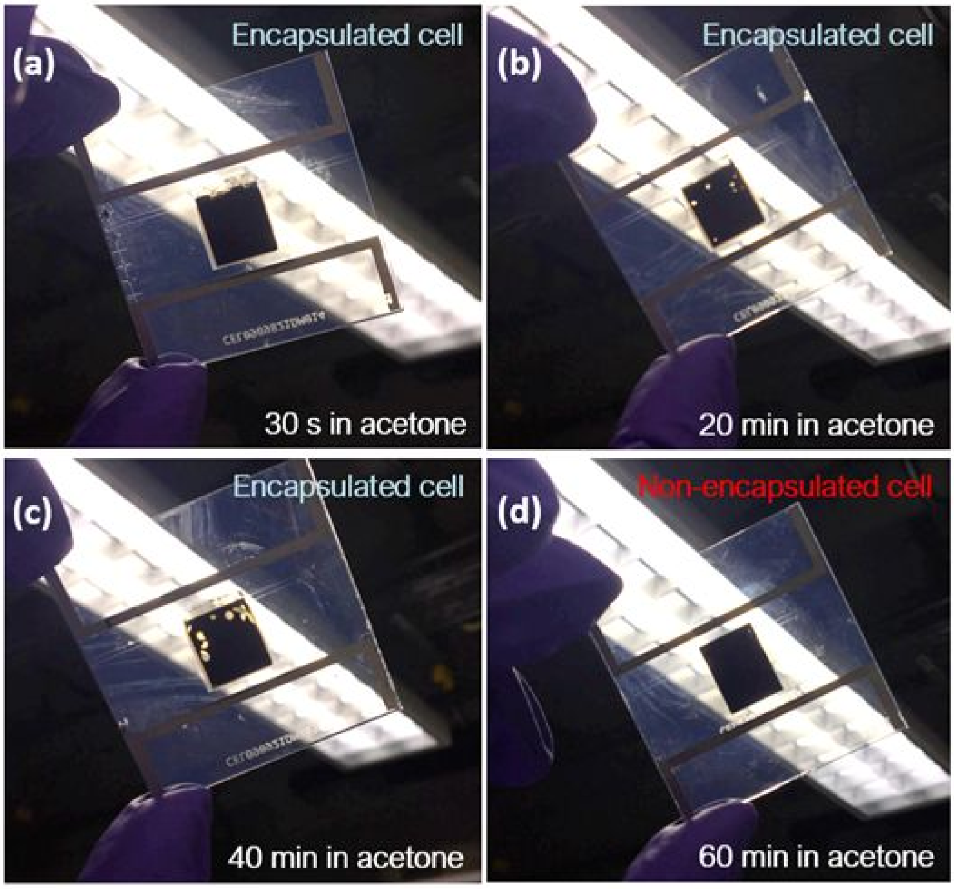

Figure S 12: Photographs showing the removal of carbon and perovskite under the influence of acetone present for different durations in encapsulated and a non-encapsulated cell

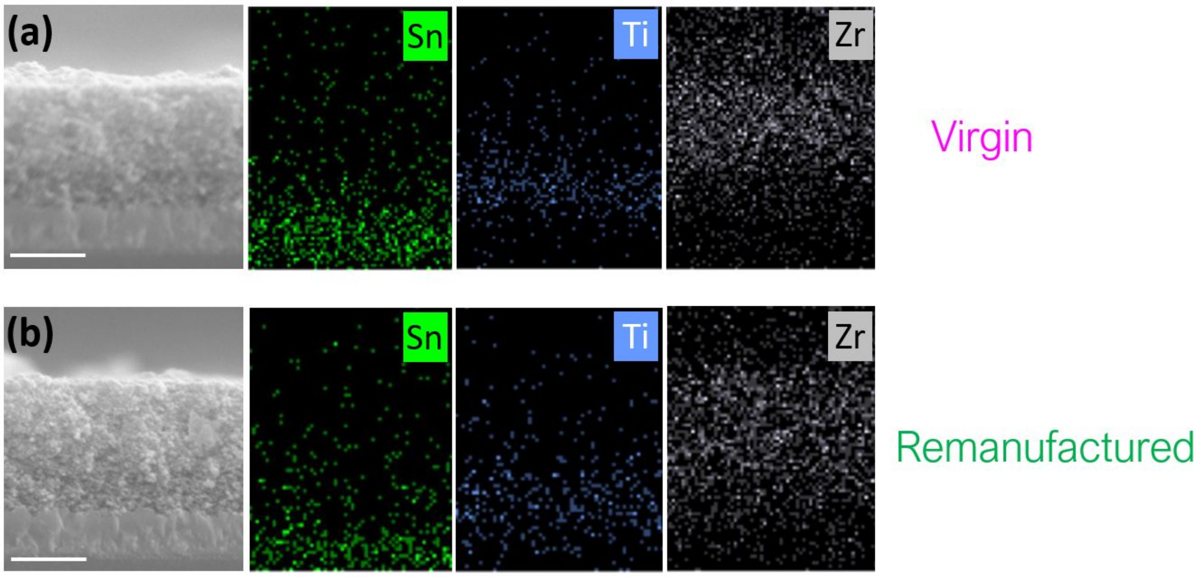

Figure S 13: SEM cross-sectional images of virgin (before remanufacturing) and remanufactured FTO/c-TiO_2/m-TiO_2/ZrO_2 stacks, as well as EDX maps of these cross-sections showing that the layer morphology and elemental composition remains unchanged before and after remanufacturing.

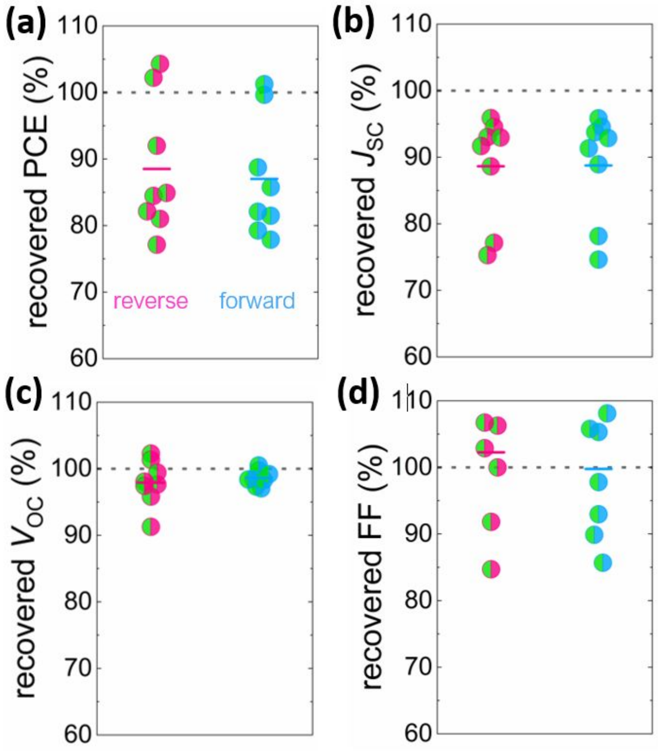

Figure S 14: Recovered (a) PCE, (b) J_{SC}, (c) V_{OC} and (d) FF after remanufacturing CPSCs, as measured from reverse and forward scans, relatively to the initial value (100%)

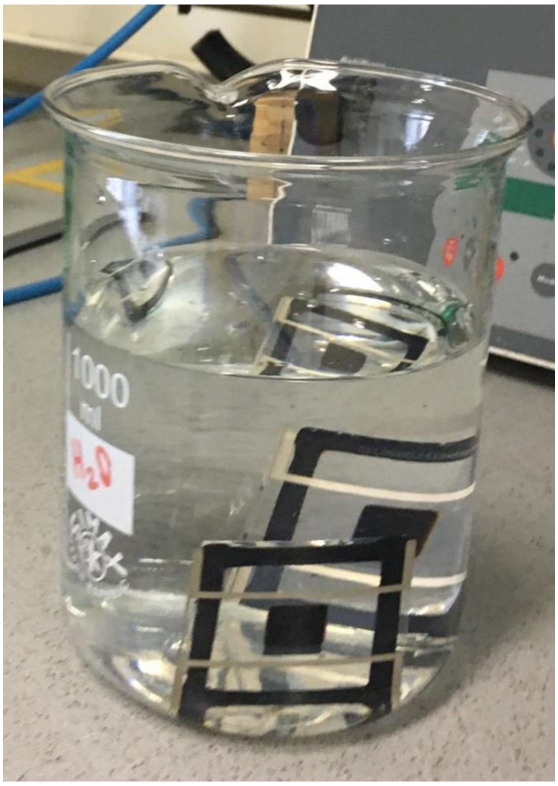

Figure S 15: Encapsulated C-PSCs submerged in water for testing water permeation

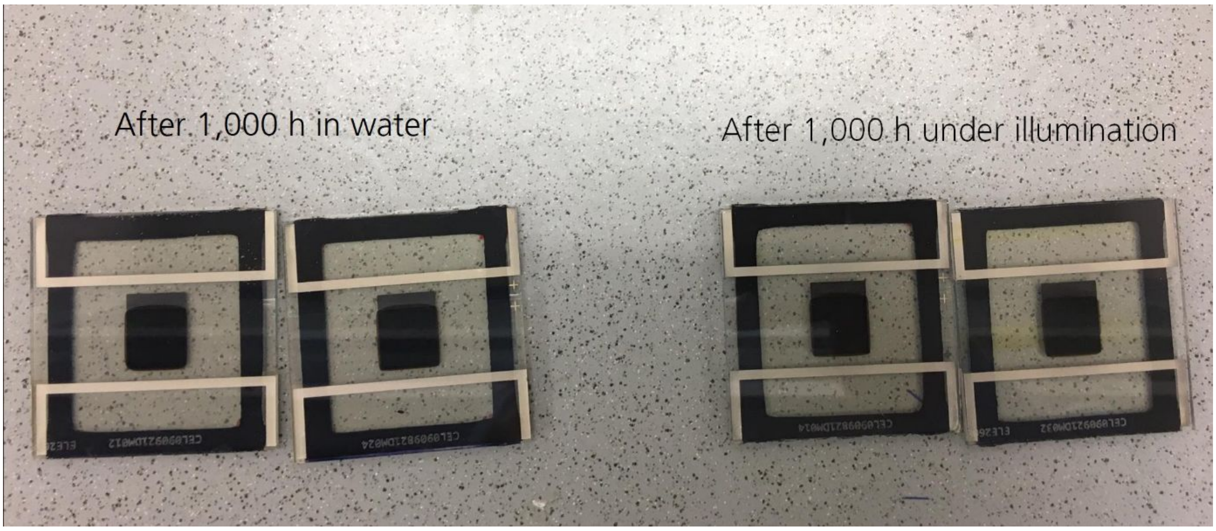

Figure S 16: Encapsulated C-PSCs after being submerged in water and undergoing a continuous illumination for 1,000 h at open-circuit, exhibiting dark appearance in the active area without loss of photoactive material

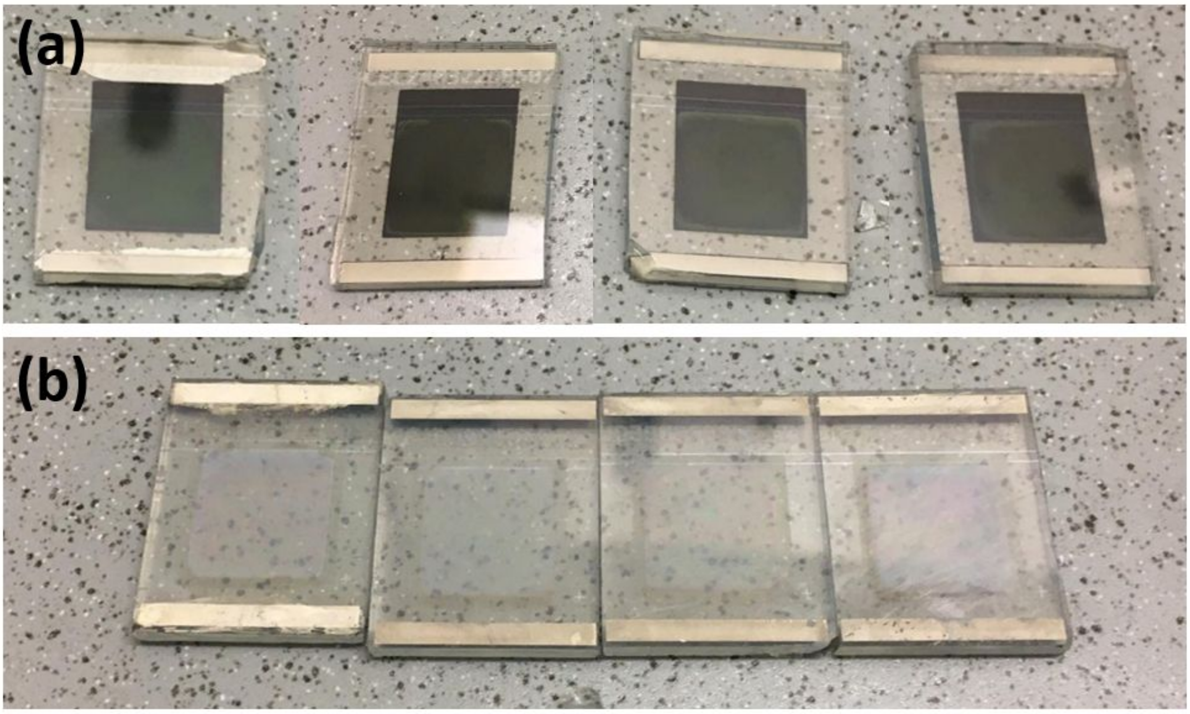

Figure S 17: (a) Degraded non-encapsulated C-PSCs and (b) same cells with carbon and degraded perovskite being removed. Note that the additional silver paste on the electrodes present in the most left sample in (a) was removed during the MA^0-liquefaction step.

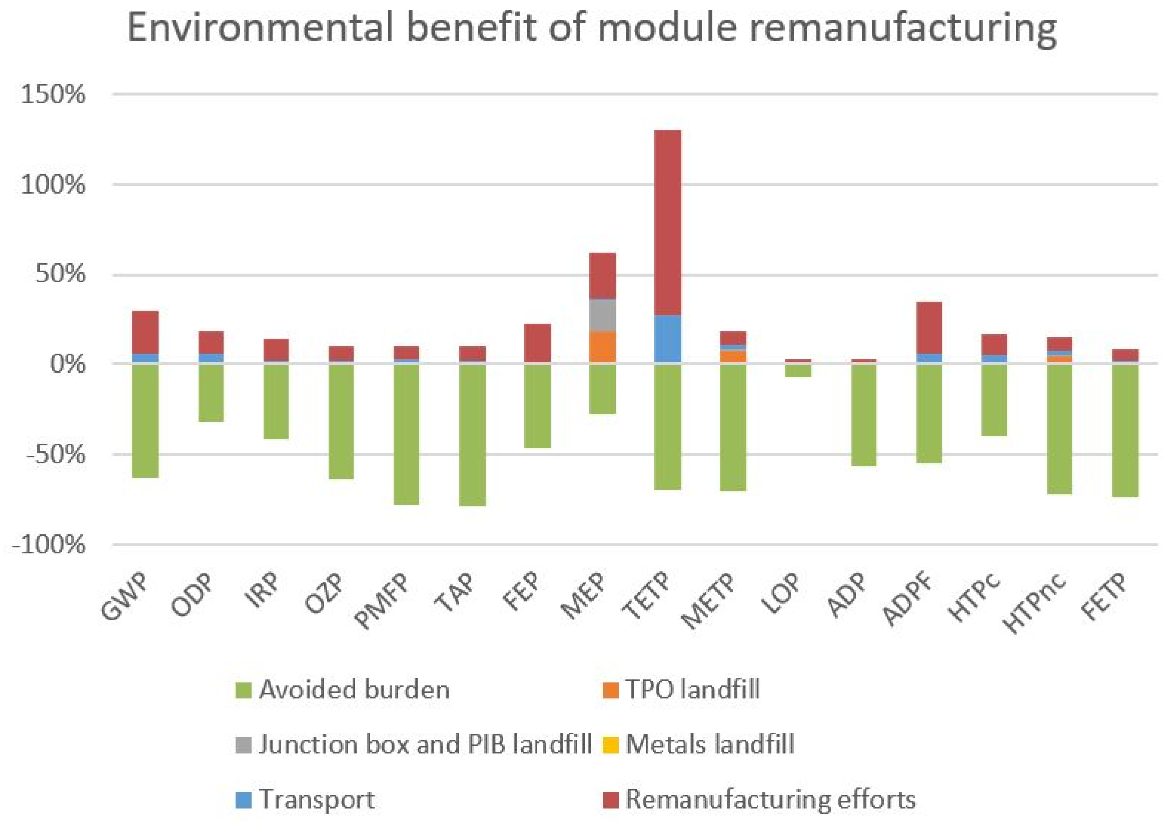

Figure S 18: Complete environmental impacts of the remanufactured PSM, relative to the virgin PSM (0%).

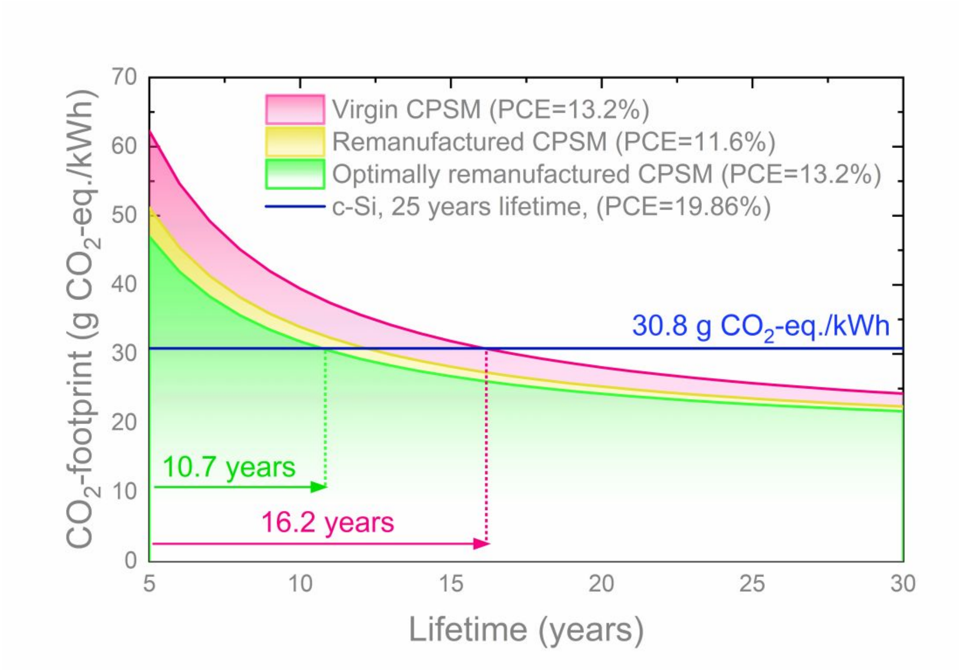

Figure S 19: CO_2-footprint of virgin and remanufactured CPSMs investigated in this work as a function of device lifetime in comparison to c-Si modules currently available on the market (as baseline)

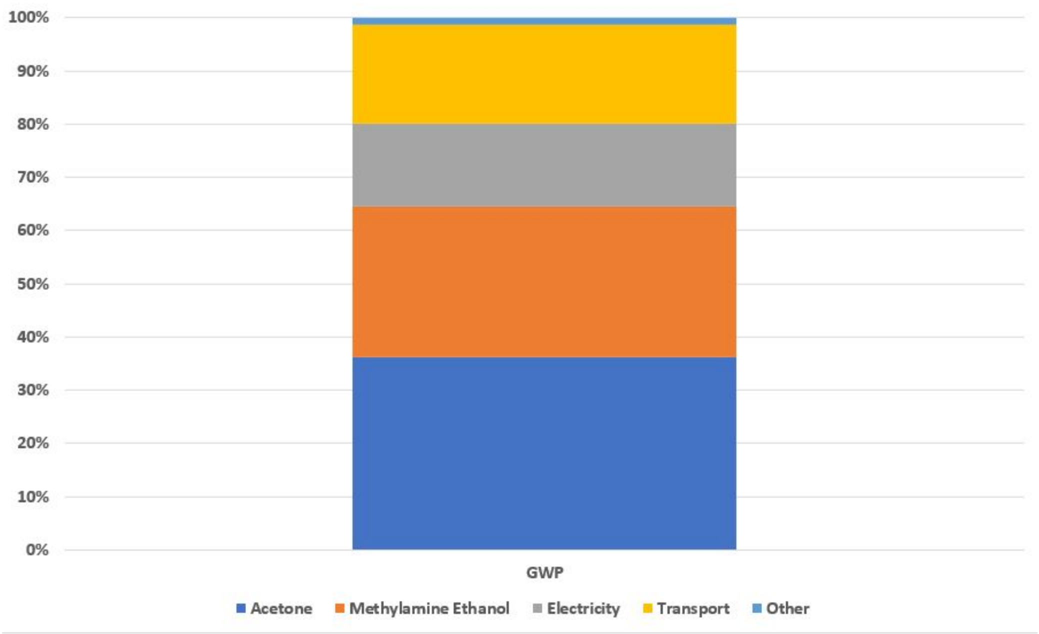

Figure S 20: Different contributions of the additional GWP originating from remanufacturing efforts, where solvents (acetone, methylamine and ethanol) account for more than 60% of it.

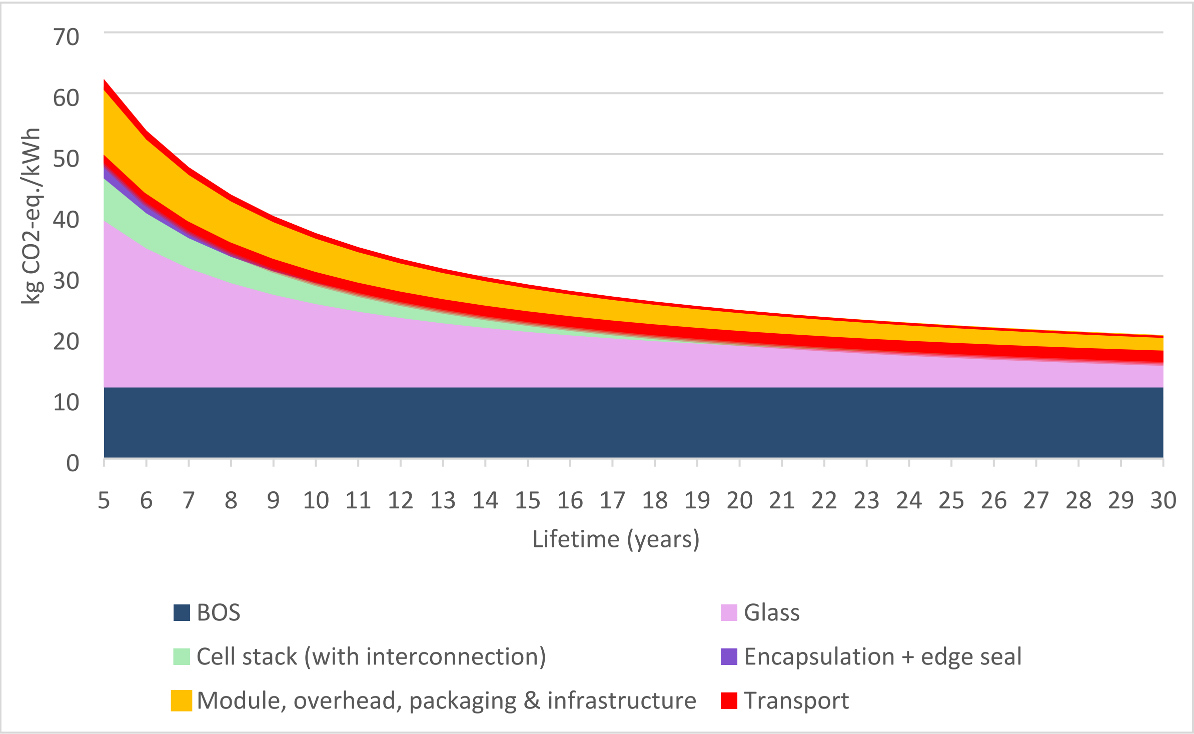

Figure S 21: GWP contributions of the different (virgin) PSM module parts as well as the BOS per kWh as a function of lifetime. The PCE used here is 13.2%.

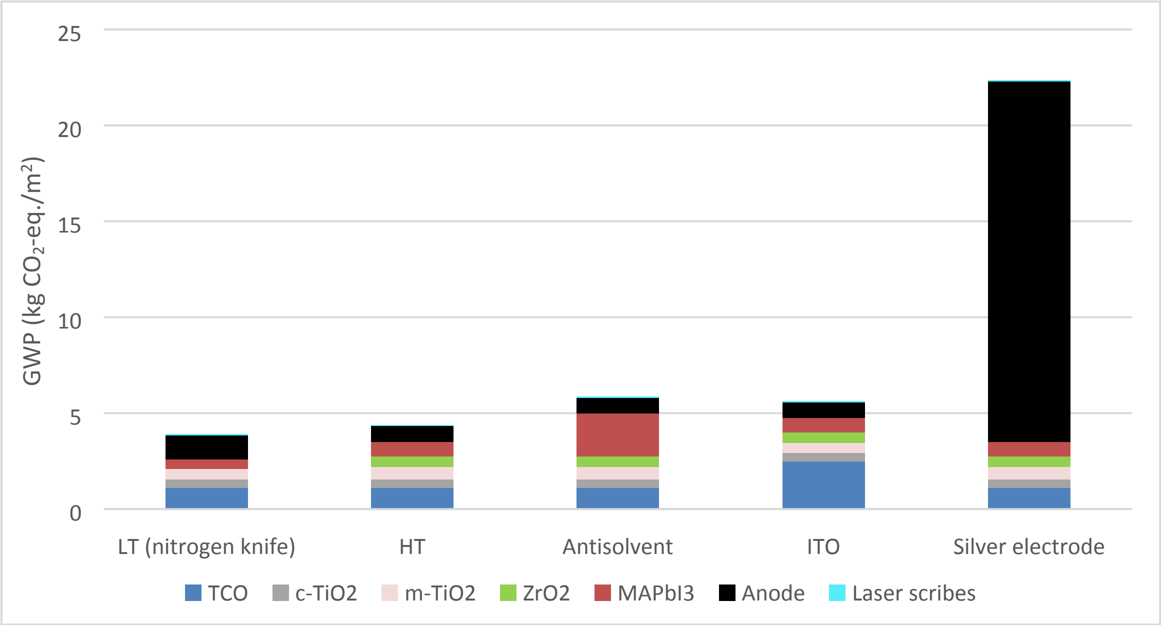

Figure S 22: GWP contributions (per m2) and totals of the PSC cell stack for different architectures

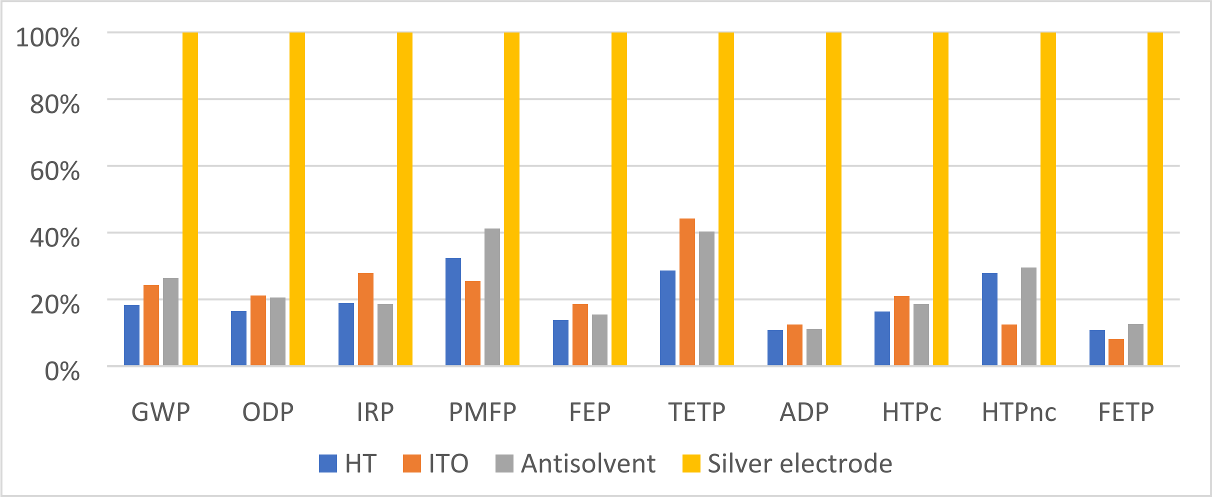

Figure S 23: Relative environmental impacts of the cell stack of different PSC architectures.

Figure S22 and S23 show the different GWP and environmental impacts (across many categories) for different PSC architectures similar to the one described in the main paper. For comparison's sake, only the impact related to the different cell layers (e.g. no glass and back sheet etc.) are shown. Note that the impacts are shown per m^2, so PCE is not (yet) taken into account.

"HT" Represents the 'standard' module that is used throughout the paper. "LT" Represents a low-temperature module, where the carbon is deposited (after the perovskite) layer and annealed at a low temperature (120 ^oC). This module does not contain a HTL and has different thicknesses for the m-TiO_2, carbon, and the perovskite layer than the HT module. Additionally, for stability purposes, this module is assumed to receive either nitrogen (using a nitrogen knife) or antisolvent (in this case ethyl acetate) treatment after the perovskite layer deposition. The "ITO", "Antisolvent" and "Silver electrode" architectures only differ from the "HT" architecture in one layer each: the "ITO" module

has an (sputtered) ITO transparent conductive oxide layer (instead of FTO), and the "Silver electrode" has a thermally evaporated silver electrode (instead of carbon).

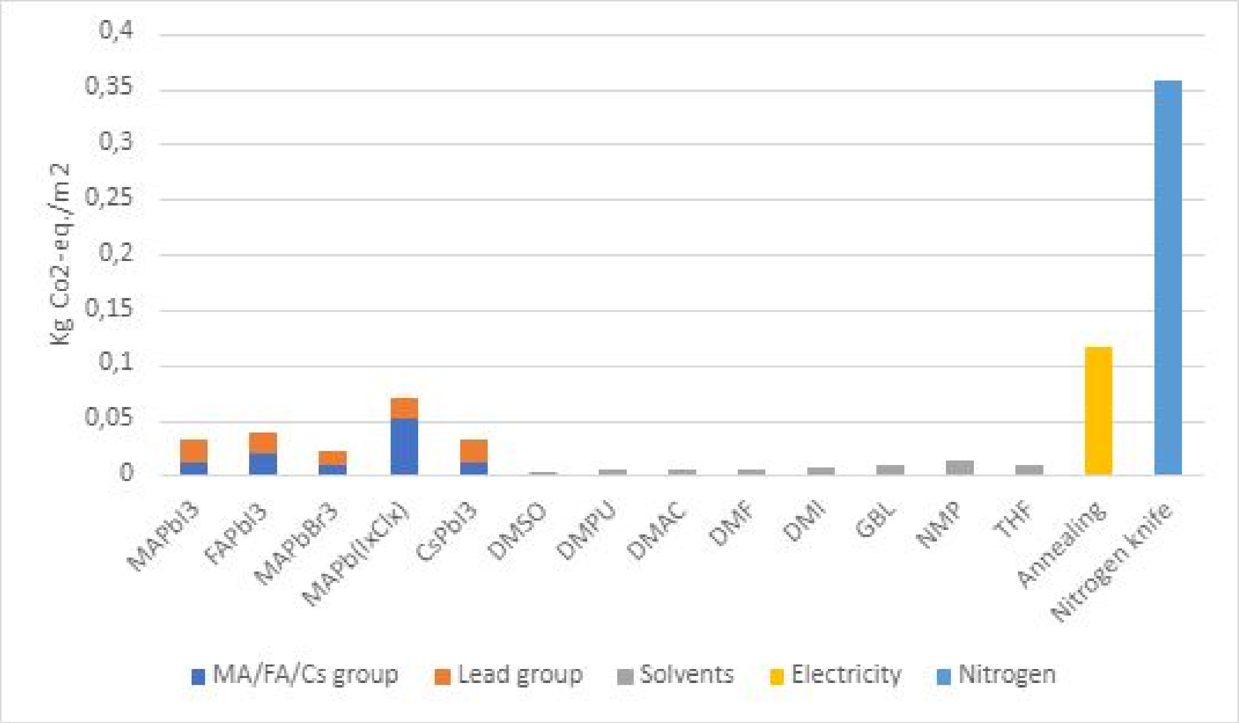

Figure S 24: GWP impacts per m² of the different perovskite absorber layer components and solvents, with the annealing part and nitrogen knife (LT) impacts of the perovskite layer deposition for comparison.

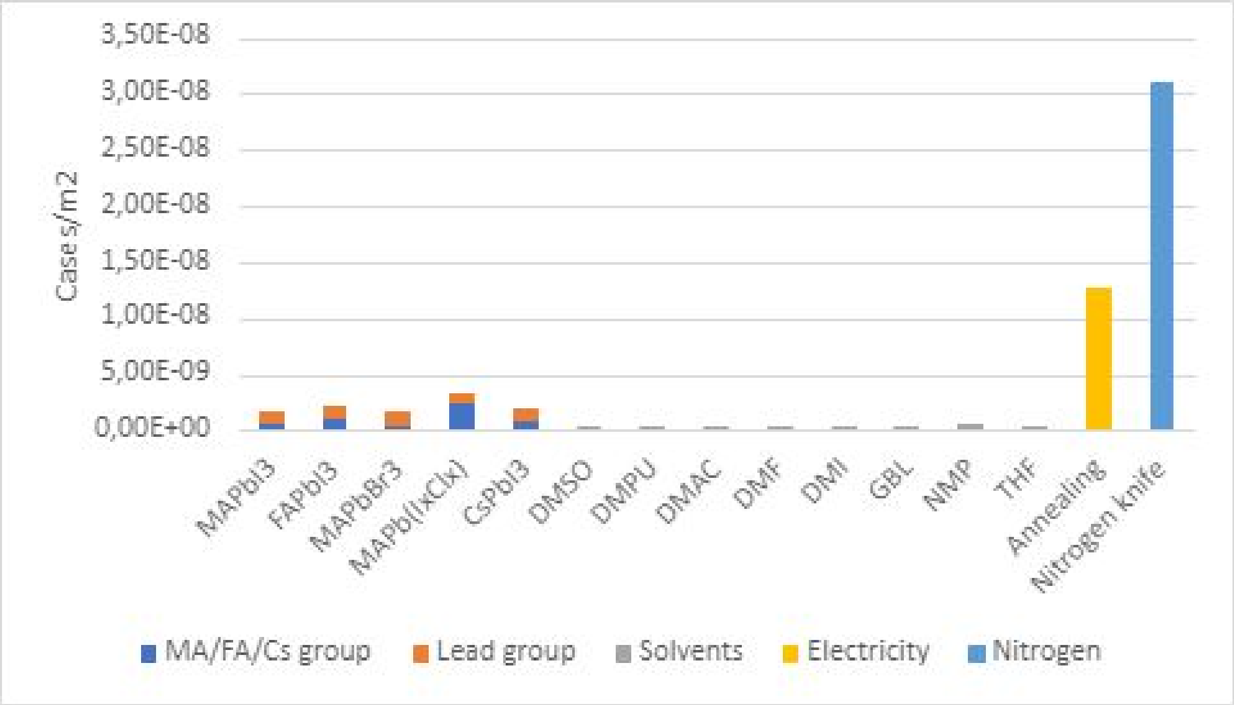

Figure S 25: Human toxicity per m² of the different perovskite absorber layer components and solvents, with the annealing part and nitrogen knife (LT) impacts of the perovskite layer deposition for comparison.

References

(1) Gaddam, S. K.; Pothu, R.; Boddula, R. Advanced polymer encapsulates for photovoltaic devices − A review. *Journal of Materiomics* **2021**, *7* (5), 920–928. DOI: 10.1016/j.jmat.2021.04.004.

(2) Kempe, M. D.; Jorgensen, G. J.; Terwilliger, K. M.; McMahon, T. J.; Kennedy, C. E.; Borek, T. T. Acetic acid production and glass transition concerns with ethylene-vinyl acetate used in photovoltaic devices. *Solar Energy Materials and Solar Cells* **2007**, *91* (4), 315–329. DOI: 10.1016/j.solmat.2006.10.009.

(3) Tracy, J.; Bosco, N.; Delgado, C.; Dauskardt, R. Durability of ionomer encapsulants in photovoltaic modules. *Solar Energy Materials and Solar Cells* **2020**, *208*, 110397.

(4) Cheacharoen, R.; Boyd, C. C.; Burkhard, G. F.; Leijtens, T.; Raiford, J. A.; Bush, K. A.; Bent, S. F.; McGehee, M. D. Encapsulating perovskite solar cells to withstand damp heat and thermal cycling. *Sustainable Energy Fuels* **2018**, *2* (11), 2398–2406. DOI: 10.1039/C8SE00250A.

(5) Adothu, B.; Bhatt, P.; Zele, S.; Oderkerk, J.; Costa, F. R.; Mallick, S. Investigation of newly developed thermoplastic polyolefin encapsulant principle properties for the c-Si PV module application. *Materials Chemistry and Physics* **2020**, *243*, 122660. DOI: 10.1016/j.matchemphys.2020.122660.

(6) Kempe, M. D.; Panchagade, D.; Reese, M. O.; Dameron, A. A. Modeling moisture ingress through polyisobutylene-based edge-seals. *Prog. Photovolt: Res. Appl.* **2015**, *23* (5), 570–581. DOI: 10.1002/pip.2465.

(7) Boyd, R. H.; Pant, P. V. K. Molecular packing and diffusion in polyisobutylene. *Macromolecules* **1991**, *24* (23), 6325–6331. DOI: 10.1021/ma00023a040.

(8) Dhere, N. G.; Wohlgemuth, J. H.; Lynn, K., Eds. *Reliability of Photovoltaic Cells, Modules, Components, and Systems III*; SPIE Proceedings; SPIE, 2010.

(9) Shi, L.; Young, T. L.; Kim, J.; Sheng, Y.; Wang, L.; Chen, Y.; Feng, Z.; Keevers, M. J.; Hao, X.; Verlinden, P. J.; Green, M. A.; Ho-Baillie, A. W. Y. Accelerated Lifetime Testing of Organic-Inorganic Perovskite Solar Cells Encapsulated by Polyisobutylene. *ACS applied materials & interfaces* **2017**, *9* (30), 25073–25081. DOI: 10.1021/acsami.7b07625. Published Online: Jul. 24, 2017.

(10) ISO. *ISO, "ISO 14044:2006. Environmental management - Life cycle assessment - Requirements and guidelines*. https://www.iso.org/cms/render/live/en/sites/isoorg/contents/data/standard/03/74/37456.html (accessed 2021-07-21).

(11) ISO. *ISO 14040:2006. Environmental management - Life cycle assessment - Principles and framework.*, 2006. https://www.iso.org/cms/render/live/en/sites/isoorg/contents/data/standard/03/74/37456.html (accessed 2021-07-21).

(12) R. Frischknecht, P. Stolz, G. Heath, M. Raugei, P. Sinha, and M. de Wild-Scholten. *Methodology Guidelines on Life Cycle Assessment of Photovoltaic 2020*. https://iea-pvps.org/wp-content/uploads/2020/07/IEA_Task12_LCA_Guidelines.pdf.

(13) Simon, B.; Bachtin, K.; Kiliç, A.; Amor, B.; Weil, M. Proposal of a framework for scale-up life cycle inventory: A case of nanofibers for lithium iron phosphate cathode applications. *Integrated environmental assessment and management* **2016**, *12* (3), 465–477. DOI: 10.1002/ieam.1788. Published Online: Jun. 4, 2016.

(14) Thonemann, N.; Schulte, A.; Maga, D. How to Conduct Prospective Life Cycle Assessment for Emerging Technologies? A Systematic Review and Methodological Guidance. *Sustainability* **2020**, *12* (3), 1192. DOI: 10.3390/su12031192.

(15) Swartwout, R.; Hoerantner, M. T.; Bulović, V. Scalable Deposition Methods for Large‐area Production of Perovskite Thin Films. *Energy Environ. Mater.* **2019**, *2* (2), 119–145. DOI: 10.1002/eem2.12043.
(16) Li, Z.; Klein, T. R.; Kim, D. H.; Yang, M.; Berry, J. J.; van Hest, M. F. A. M.; Zhu, K. Scalable fabrication of perovskite solar cells. *Nat Rev Mater* **2018**, *3* (4). DOI: 10.1038/natrevmats.2018.17.
(17) Frischknecht, R.; Heath, G.; Raugei, M.; Sinha, P.; Wild-Scholten, M. de. *Methodology guidelines on life cycle assessment of photovoltaic electricity*.
(18) Mark Goedkoop, Michiel Oele, Jorrit Leijting, Tommie Ponsioen, Ellen Meijer. *Introduction to LCA with SimaPro*. https://pre-sustainability.com/files/2014/05/SimaPro8IntroductionToLCA.pdf.
(19) Chatzisideris, M. D.; Espinosa, N.; Laurent, A.; Krebs, F. C. Ecodesign perspectives of thin-film photovoltaic technologies: A review of life cycle assessment studies. *Solar Energy Materials and Solar Cells* **2016**, *156*, 2–10. DOI: 10.1016/j.solmat.2016.05.048.
(20) Amarakoon, S.; Vallet, C.; Curran, M. A.; Haldar, P.; Metacarpa, D.; Fobare, D.; Bell, J. Life cycle assessment of photovoltaic manufacturing consortium (PVMC) copper indium gallium (di)selenide (CIGS) modules. *Int J Life Cycle Assess* **2018**, *23* (4), 851–866. DOI: 10.1007/s11367-017-1345-4.
(21) Lunardi, M.; Alvarez-Gaitan, J.; Bilbao, J.; Corkish, R. Comparative Life Cycle Assessment of End-of-Life Silicon Solar Photovoltaic Modules. *Applied Sciences* **2018**, *8* (8), 1396. DOI: 10.3390/app8081396.
(22) Goe, M.; Gaustad, G. Estimating direct climate impacts of end-of-life solar photovoltaic recovery. *Solar Energy Materials and Solar Cells* **2016**, *156*, 27–36. DOI: 10.1016/j.solmat.2016.04.025.
(23) Stamford, L.; Azapagic, A. Environmental Impacts of Photovoltaics: The Effects of Technological Improvements and Transfer of Manufacturing from Europe to China. *Energy Technol.* **2018**, *6* (6), 1148–1160. DOI: 10.1002/ente.201800037.
(24) Desideri, U.; Proietti, S.; Zepparelli, F.; Sdringola, P.; Bini, S. Life Cycle Assessment of a ground-mounted 1778kWp photovoltaic plant and comparison with traditional energy production systems. *Applied Energy* **2012**, *97*, 930–943. DOI: 10.1016/j.apenergy.2012.01.055.
(25) Müller, A.; Friedrich, L.; Reichel, C.; Herceg, S.; Mittag, M.; Neuhaus, D. H. A comparative life cycle assessment of silicon PV modules: Impact of module design, manufacturing location and inventory. *Solar Energy Materials and Solar Cells* **2021**, *230*, 111277. DOI: 10.1016/j.solmat.2021.111277.
(26) Gomez Trillos, J. C.; Brand, U.; Vogt, T. Life Cycle Assessment of Perovskite Tandem Solar Cells **2020**.
(27) M. Lunardi, M.; Alvarez-Gaitan, J. P.; Chang, N. L.; Corkish, R. Life cycle assessment on PERC solar modules. *Solar Energy Materials and Solar Cells* **2018**, *187*, 154–159. DOI: 10.1016/j.solmat.2018.08.004.
(28) Solargis. *"Global Solar Atlas"*. https://globalsolaratlas.info/map?c=48.01542,7.814369,11&s= 48.009219,7.876511&m=site.
(29) Cai, M.; Wu, Y.; Chen, H.; Yang, X.; Qiang, Y.; Han, L. Cost-Performance Analysis of Perovskite Solar Modules. *Advanced science (Weinheim, Baden-Wurttemberg, Germany)* **2017**, *4* (1), 1600269. DOI: 10.1002/advs.201600269. Published Online: Sep. 15, 2016.
(30) Chang, N. L.; Yi Ho-Baillie, A. W.; Basore, P. A.; Young, T. L.; Evans, R.; Egan, R. J. A manufacturing cost estimation method with uncertainty analysis and its application to perovskite on glass photovoltaic modules. *Prog. Photovolt: Res. Appl.* **2017**, *25* (5), 390–405. DOI: 10.1002/pip.2871.

(31) Song, Z.; McElvany, C. L.; Phillips, A. B.; Celik, I.; Krantz, P. W.; Watthage, S. C.; Liyanage, G. K.; Apul, D.; Heben, M. J. A technoeconomic analysis of perovskite solar module manufacturing with low-cost materials and techniques. *Energy Environ. Sci.* **2017**, *10* (6), 1297–1305. DOI: 10.1039/C7EE00757D.
(32) Jung, E. H.; Jeon, N. J.; Park, E. Y.; Moon, C. S.; Shin, T. J.; Yang, T.-Y.; Noh, J. H.; Seo, J. Efficient, stable and scalable perovskite solar cells using poly(3-hexylthiophene). *Nature* **2019**, *567* (7749), 511–515. DOI: 10.1038/s41586-019-1036-3. Published Online: Mar. 27, 2019.
(33) Galagan, Y.; Coenen, E. W. C.; Verhees, W. J. H.; Andriessen, R. Towards the scaling up of perovskite solar cells and modules. *J. Mater. Chem. A* **2016**, *4* (15), 5700–5705. DOI: 10.1039/C6TA01134A.
(34) Rakocevic, L.; Gehlhaar, R.; Merckx, T.; Qiu, W.; Paetzold, U. W.; Fledderus, H.; Poortmans, J. Interconnection optimization for highly efficient perovskite modules. *IEEE Journal of Photovoltaics* **2016**, *7* (1), 404–408.
(35) Castriotta, L. A.; Matteocci, F.; Vesce, L.; Cinà, L.; Agresti, A.; Pescetelli, S.; Ronconi, A.; Löffler, M.; Stylianakis, M. M.; Di Giacomo, F.; Mariani, P.; Stefanelli, M.; Speller, E. M.; Alfano, A.; Paci, B.; Generosi, A.; Di Fonzo, F.; Petrozza, A.; Rellinghaus, B.; Kymakis, E.; Di Carlo, A. Air-Processed Infrared-Annealed Printed Methylammonium-Free Perovskite Solar Cells and Modules Incorporating Potassium-Doped Graphene Oxide as an Interlayer. *ACS applied materials & interfaces* **2021**, *13* (10), 11741–11754. DOI: 10.1021/acsami.0c18920. Published Online: Mar. 2, 2021.
(36) Di Giacomo, F.; Castriotta, L. A.; Kosasih, F. U.; Di Girolamo, D.; Ducati, C.; Di Carlo, A. Upscaling Inverted Perovskite Solar Cells: Optimization of Laser Scribing for Highly Efficient Mini-Modules. *Micromachines* **2020**, *11* (12). DOI: 10.3390/mi11121127. Published Online: Dec. 20, 2020.
(37) Kempe, M. D.; Panchagade, D.; Reese, M. O.; Dameron, A. A. Modeling moisture ingress through polyisobutylene‐based edge‐seals. *Prog. Photovolt: Res. Appl.* **2015**, *23* (5), 570–581.
(38) Stolz, P.; Frischknecht, R.; Wambach, K.; Sinha, P.; Heath, G. Life cycle assessment of current photovoltaic module recycling. *IEA PVPS Task 12, International Energy Agency Power Systems Programme, Report IEA-PVPS T12* **2017**, *13*, 2018.
(39) Jordan, D. C.; Kurtz, S. R.; VanSant, K.; Newmiller, J. Compendium of photovoltaic degradation rates. *Prog. Photovolt: Res. Appl.* **2016**, *24* (7), 978–989. DOI: 10.1002/pip.2744.
(40) Huld, T.; Friesen, G.; Skoczek, A.; Kenny, R. P.; Sample, T.; Field, M.; Dunlop, E. D. A power-rating model for crystalline silicon PV modules. *Solar Energy Materials and Solar Cells* **2011**, *95* (12), 3359–3369.
(41) Lia, F. de; Castello, S.; Abenante, L. Efficiency degradation of c-silicon photovoltaic modules after 22-year continuous field exposure. In *3rd World Conference onPhotovoltaic Energy Conversion, 2003. Proceedings of*, 2003, Vol. 2; 2105-2108 Vol.2.
(42) Jordan, D. C.; Kurtz, S. R. Photovoltaic Degradation Rates-an Analytical Review. *Prog. Photovolt: Res. Appl.* **2013**, *21* (1), 12–29. DOI: 10.1002/pip.1182.
(43) Jordan, D. C.; Silverman, T. J.; Sekulic, B.; Kurtz, S. R. PV degradation curves: non‐linearities and failure modes. *Prog. Photovolt: Res. Appl.* **2017**, *25* (7), 583–591. DOI: 10.1002/pip.2835.
(44) European Commission. Joint Research Centre. *Photovoltaics: technology development report 2020*; Publications Office, 2020. DOI: 10.2760/827685.
(45) Khalifa, A.; Shafie, S.; Hasan, W. Z.; Lim, H. N.; Rusop, M.; Pandey, S. S.; Vats, A. K.; AlSultan, H. A.; Samaila, B. Comprehensive performance analysis of dye-sensitized solar cells using single layer TiO2 photoanode deposited using screen printing technique. *Optik* **2020**, *223*, 165595. DOI: 10.1016/j.ijleo.2020.165595.

(46) Fraunhofer ISE, PSE Projects GmbH. *Photovoltaics report*. https://www.ise.fraunhofer.de/content/dam/ise/de/documents/publications/studies/Photovoltaics-Report.pdf.
(47) Mainzer, K.; Killinger, S.; McKenna, R.; Fichtner, W. Assessment of rooftop photovoltaic potentials at the urban level using publicly available geodata and image recognition techniques. *Solar Energy* **2017**, *155*, 561–573. DOI: 10.1016/j.solener.2017.06.065.
(48) First Solar. *Series 6 - Module datasheet*. https://www.firstsolar.com/-/media/First-Solar/Technical-Documents/Series-6-.
(49) Virtuani, A.; Pavanello, D.; Friesen, G. Overview of temperature coefficients of different thin film photovoltaic technologies. In *25th European photovoltaic solar energy conference and exhibition/5th World conference on photovoltaic energy conversion*; pp 3–83.
(50) Vidal, R.; Alberola ‐ Borràs, J.-A.; Sánchez ‐ Pantoja, N.; Mora ‐ Seró, I. Comparison of Perovskite Solar Cells with other Photovoltaics Technologies from the Point of View of Life Cycle Assessment. *Adv Energy Sustain Res* **2021**, *2* (5), 2000088. DOI: 10.1002/aesr.202000088.
(51) Geisler, G.; Hofstetter, T. B.; Hungerbühler, K. Production of fine and speciality chemicals: procedure for the estimation of LCIs. *Int J Life Cycle Assess* **2004**, *9* (2), 101–113. DOI: 10.1007/BF02978569.
(52) Piccinno, F.; Hischier, R.; Seeger, S.; Som, C. From laboratory to industrial scale: a scale-up framework for chemical processes in life cycle assessment studies. *Journal of Cleaner Production* **2016**, *135*, 1085–1097. DOI: 10.1016/j.jclepro.2016.06.164.
(53) Bai, Y.; Muralidharan, N.; Li, J.; Essehli, R.; Belharouak, I. Sustainable Direct Recycling of Lithium ‐ Ion Batteries via Solvent Recovery of Electrode Materials. *ChemSusChem* **2020**, *13* (21), 5664–5670. DOI: 10.1002/cssc.202001479.
(54) Capello, C.; Hellweg, S.; Badertscher, B.; Hungerbühler, K. Life-cycle inventory of waste solvent distillation: statistical analysis of empirical data. *Environmental science & technology* **2005**, *39* (15), 5885–5892. DOI: 10.1021/es048114o.
(55) Parvatker, A. G.; Tunceroglu, H.; Sherman, J. D.; Coish, P.; Anastas, P.; Zimmerman, J. B.; Eckelman, M. J. Cradle-to-Gate Greenhouse Gas Emissions for Twenty Anesthetic Active Pharmaceutical Ingredients Based on Process Scale-Up and Process Design Calculations. *ACS Sustainable Chem. Eng.* **2019**, *7* (7), 6580–6591. DOI: 10.1021/acssuschemeng.8b05473.
(56) Osipov, E.; Telyakov, E.; Ponikarov, S. Coupled Simulation of a Vacuum Creation System and a Rectification Column Block. *Processes* **2020**, *8* (11), 1333. DOI: 10.3390/pr8111333.
(57) Green, D. W. Perry's chemical engineers'. *Handbook–seventh Edition–Sections* **2008**, 5–12.
(58) EQUIPMENT, PROCESS DESIGN OF VACUUM. Engineering Standard. *Quest Technical Publication PUB* **1993**, *77385*.
(59) Leybold. *Dry Compressing Vacuum Pumps*. https://www.leyboldproducts.com/media/pdf/43/39/a0/CP_020_Dry_Compressing_Vacuum_Pumps_EN.pdf.
(60) Goetzler, W.; Zogg, R.; Young, J.; Johnson, C. *Energy Savings Potential and RD&D Opportunities for NonVapor-Energy Savings Potential and RD&D Opportunities for Non-Vapor-Compression Compression HVAC Technologies*. https://www.energy.gov/sites/prod/files/2014/03/f12/Non-Vapor%20Compression%20HVAC%20Report.pdf.
(61) Althaus, H. J.; Chudacoff, M.; Hischier, R.; Jungbluth, N.; Osses, M.; Primas, A. Life cycle inventories of chemicals. *Ecoinvent report* **2007**, *2*.
(62) Gendorf Chemiepark. *Unwelt Erklärung 2017*. https://www.gendorf.de/-/media/Internet/chemiepark_gendorf_de/Downloads/Nachbarschaft/Umwelterklaerung_2017_final.ashx?la=de-DE https://www.gendorf.de/-

/media/Internet/chemiepark_gendorf_de/Downloads/Nachbarschaft/Umwelterklaerung_2017_final.ashx?la=de-DE.
(63) Frischknecht, R.; Jungbluth, N.; Althaus, H.-J.; Hischier, R.; Doka, G.; Dones, R.; Heck, T.; Hellweg, S.; Wernet, G.; Nemecek, T. *Overview and methodology. Data v2. 0 (2007). Ecoinvent report No. 1*.
(64) Aden, N.; Stork, M.; Chang Kylee. *Barriers, Challenges, and Opportunities for Chemical Companies to Set Science-Based Targets*. https://sciencebasedtargets.org/resources/files/SBTi-Chemicals-Scoping-Document-12.2020.pdf.
(65) Hassim, M. H.; Hurme, M.; Amyotte, P. R.; Khan, F. I. Fugitive emissions in chemical processes: The assessment and prevention based on inherent and add-on approaches. *Journal of Loss Prevention in the Process Industries* **2012**, *25* (5), 820–829. DOI: 10.1016/j.jlp.2012.03.004.
(66) Mitchell Scientific, Inc., RTI International. *Methods for Estimating Air Emissions from Chemical Manufacturing Facilities*. https://www.epa.gov/sites/default/files/2015-08/documents/ii16_aug2007final.pdf.
(67) Hischier, R.; Hellweg, S.; Capello, C.; Primas, A. Establishing Life Cycle Inventories of Chemicals Based on Differing Data Availability (9 pp). *Int J Life Cycle Assess* **2005**, *10* (1), 59–67. DOI: 10.1065/lca2004.10.181.7.
(68) US Environmental Protection Agency. *Consolidated List of Lists under Consolidated List of Lists under EPCRA/CERCLA/CAA §112(r) (April 2022 Version)*. https://www.epa.gov/system/files/documents/2022-04/list_of_lists_compiled_april-2022.pdf.
(69) Bazzanella, A.; Ausfelder, F. *Low carbon energy and feedstock for the European chemical industry: Technology Study*; DECHEMA, Gesellschaft für Chemische Technik und Biotechnologie eV, 2017.
(70) Dunn, A. L.; Payne, A.; Clark, P. R.; McKay, C.; Williams, G. D.; Wheelhouse, K.; Arendt, K.; Dixon, F.; Shilcrat, S. Process Safety in the Pharmaceutical Industry: A Selection of Illustrative Case Studies. *J. Chem. Educ.* **2021**, *98* (1), 175–182. DOI: 10.1021/acs.jchemed.0c00115.
(71) Hur, J.; Moon, I. Novel Ethylene Oxide Gas Recovery System via Hydrolysis in the Dimethyl Carbonate and Monoethylene Glycol Production Process. *Ind. Eng. Chem. Res.* **2020**, *59* (7), 3091–3096. DOI: 10.1021/acs.iecr.9b06344.
(72) Alberola-Borràs, J.-A.; Baker, J. A.; Rossi, F. de; Vidal, R.; Beynon, D.; Hooper, K. E. A.; Watson, T. M.; Mora-Seró, I. Perovskite Photovoltaic Modules: Life Cycle Assessment of Pre-industrial Production Process. *iScience* **2018**, *9*, 542–551. DOI: 10.1016/j.isci.2018.10.020. Published Online: Nov. 14, 2018.
(73) Espinosa, N.; Serrano-Luján, L.; Urbina, A.; Krebs, F. C. Solution and vapour deposited lead perovskite solar cells: Ecotoxicity from a life cycle assessment perspective. *Solar Energy Materials and Solar Cells* **2015**, *137*, 303–310.
(74) Gong, J.; Darling, S. B.; You, F. Perovskite photovoltaics: life-cycle assessment of energy and environmental impacts. *Energy Environ. Sci.* **2015**, *8* (7), 1953–1968. DOI: 10.1039/C5EE00615E.
(75) Alberola-Borràs, J.-A.; Vidal, R.; Mora-Seró, I. Evaluation of multiple cation/anion perovskite solar cells through life cycle assessment. *Sustainable Energy Fuels* **2018**, *2* (7), 1600–1609. DOI: 10.1039/C8SE00053K.
(76) Turton, R.; Bailie, R. C.; Whiting, W. B.; Shaeiwitz, J. A. *Analysis, synthesis and design of chemical processes*; Pearson Education, 2008.
(77) Calabrese, G. S.; Pissavini, S. From batch to continuous flow processing in chemicals manufacturing. *AIChE journal* **2011**, *57* (4), 828–834.

(78) Khalifa, S. A.; Spatari, S.; Fafarman, A. T.; Baxter, J. B. Environmental Sustainability of Mixed Cation Perovskite Materials in Photovoltaics Manufacturing. *ACS Sustainable Chem. Eng.* **2020**, *8* (44), 16537–16548. DOI: 10.1021/acssuschemeng.0c05619.
(79) Wakamiya, A.; Endo, M.; Sasamori, T.; Tokitoh, N.; Ogomi, Y.; Hayase, S.; Murata, Y. Reproducible fabrication of efficient perovskite-based solar cells: X-ray crystallographic studies on the formation of CH3NH3PbI3 layers. *Chemistry Letters* **2014**, *43* (5), 711–713.
(80) Lee, C.; Shin, Y.; Jeon, G. G.; Kang, D.; Jung, J.; Jeon, B.; Park, J.; Kim, J.; Yoon, S. J. Cost-efficient, Effect of Low-Quality PbI2 Purification to Enhance Performances of Perovskite Quantum Dots and Perovskite Solar Cells. *Energies* **2021**, *14* (1), 201. DOI: 10.3390/en14010201.
(81) Cohen, B.-E.; Gamliel, S.; Etgar, L. Parameters influencing the deposition of methylammonium lead halide iodide in hole conductor free perovskite-based solar cells. *APL Materials* **2014**, *2* (8), 81502. DOI: 10.1063/1.4885548.
(82) Billstrand, B. M.; Bian, K.; Alarid, L. J.; Schunk, H. C.; Fan, H. *Synthesis of Lead Iodide Perovskites for Solar Cell Application*.
(83) Kyoto University. Method for producing high-efficiency perovskite solar cell.
(84) Schlessinger, G. G. *Inorganic laboratory preparations*; Chemical Publishing Company, 1962.
(85) Klein, E.; Lesyuk, R.; Klinke, C. Insights into the formation mechanism of two-dimensional lead halide nanostructures. *Nanoscale* **2018**, *10* (9), 4442–4451. DOI: 10.1039/C7NR09564C.
(86) Sigma-Aldrich. *Lead(II)-Chloride (99.999% trace metals basis) - Product description*. https://www.sigmaaldrich.com/DE/de/product/aldrich/203572.
(87) Surabhi, R.; Bhat, K.; Batra, A.; Chilvery, A.; Aggarwal, M. Synthesis, purification, crystal growth and characterization of Lead Iodide (PbI2) purified by a low-temperature technique. *Advanced Science, Engineering and Medicine* **2014**, *6* (12), 1269–1273.
(88) Khalaph, K. A.; Shanan, Z. J.; Jafar, A. M.; Al-Attar, F. M. Structural and Optical Properties of PbI_2 Thin Films to Fabricate Perovskite Solar Cells. *DDF* **2020**, *398*, 140–146. DOI: 10.4028/www.scientific.net/DDF.398.140.
(89) Kim, H.-S.; Lee, C.-R.; Im, J.-H.; Lee, K.-B.; Moehl, T.; Marchioro, A.; Moon, S.-J.; Humphry-Baker, R.; Yum, J.-H.; Moser, J. E.; Grätzel, M.; Park, N.-G. Lead iodide perovskite sensitized all-solid-state submicron thin film mesoscopic solar cell with efficiency exceeding 9%. *Sci Rep* **2012**, *2* (1), 591. DOI: 10.1038/srep00591. Published Online: Aug. 21, 2012.
(90) Cai, B.; Xing, Y.; Yang, Z.; Zhang, W.-H.; Qiu, J. High performance hybrid solar cells sensitized by organolead halide perovskites. *Energy Environ. Sci.* **2013**, *6* (5), 1480. DOI: 10.1039/c3ee40343b.
(91) Kim, H.-S.; Lee, J.-W.; Yantara, N.; Boix, P. P.; Kulkarni, S. A.; Mhaisalkar, S.; Grätzel, M.; Park, N.-G. High efficiency solid-state sensitized solar cell-based on submicrometer rutile TiO2 nanorod and CH3NH3PbI3 perovskite sensitizer. *Nano letters* **2013**, *13* (6), 2412–2417. DOI: 10.1021/nl400286w. Published Online: May. 16, 2013.
(92) Etgar, L.; Gao, P.; Xue, Z.; Peng, Q.; Chandiran, A. K.; Liu, B.; Nazeeruddin, M. K.; Grätzel, M. Mesoscopic CH3NH3PbI3/TiO2 heterojunction solar cells. *Journal of the American Chemical Society* **2012**, *134* (42), 17396–17399. DOI: 10.1021/ja307789s. Published Online: Oct. 11, 2012.
(93) Heo, J. H.; Im, S. H.; Noh, J. H.; Mandal, T. N.; Lim, C.-S.; Chang, J. A.; Lee, Y. H.; Kim, H.; Sarkar, A.; Nazeeruddin, M. K.; Grätzel, M.; Seok, S. I. Efficient inorganic–organic hybrid heterojunction solar cells containing perovskite compound and polymeric hole conductors. *Nature Photon* **2013**, *7* (6), 486–491. DOI: 10.1038/nphoton.2013.80.
(94) Snaith, H. J. Perovskites: The Emergence of a New Era for Low-Cost, High-Efficiency Solar Cells. *J. Phys. Chem. Lett.* **2013**, *4* (21), 3623–3630. DOI: 10.1021/jz4020162.

(95) Noh, J. H.; Im, S. H.; Heo, J. H.; Mandal, T. N.; Seok, S. I. Chemical management for colorful, efficient, and stable inorganic-organic hybrid nanostructured solar cells. *Nano letters* **2013**, *13* (4), 1764–1769. DOI: 10.1021/nl400349b. Published Online: Mar. 21, 2013.
(96) Im, J.-H.; Lee, C.-R.; Lee, J.-W.; Park, S.-W.; Park, N.-G. 6.5% efficient perovskite quantum-dot-sensitized solar cell. *Nanoscale* **2011**, *3* (10), 4088–4093.
(97) Fujifilm. *Methylammonium iodide*:*Laboratory Chemicals-FUJIFILM Wako Chemicals U.S.A. Corporation*. https://labchem-wako.fujifilm.com/us/product/detail/W01W0113-1826.html.
(98) Borun New Material. *99.5%FAI, CH(NH2)2I, Formamidinium Iodide manufacturers and suppliers in China*. http://www.chemborun.com/products/99-5-FAI-CH-NH2-2I-Formamidinium-Iodide-2336269.html.
(99) American Elements. *Methylammonium Bromide*. https://www.americanelements.com/methylammonium-bromide-6876-37-5.
(100) Ottoboni, S.; Shahid, M.; Steven, C.; Coleman, S.; Meehan, E.; Barton, A.; Firth, P.; Sutherland, R.; Price, C. J. Developing a Batch Isolation Procedure and Running It in an Automated Semicontinuous Unit: AWL CFD25 Case Study. *Organic process research & development* **2020**, *24* (4), 520–539. DOI: 10.1021/acs.oprd.9b00512. Published Online: Mar. 26, 2020.
(101) Valastro, S.; Smecca, E.; Sanzaro, S.; Giannazzo, F.; Deretzis, I.; La Magna, A.; Numata, Y.; Jena, A. K.; Miyasaka, T.; Gagliano, A.; Alberti, A. Improved Electrical and Structural Stability in HTL-Free Perovskite Solar Cells by Vacuum Curing Treatment. *Energies* **2020**, *13* (15), 3953. DOI: 10.3390/en13153953.
(102) Moreno Ruiz, E.; Valsasina, L.; Brunner, F.; Symeonidis, A.; FitzGerald, D.; Treyer, K.; Bourgault, G.; Wernet, G. Documentation of changes implemented in ecoinvent database v3. 3. *Ecoinvent: Zürich, Switzerland* **2016**.
(103) K. Lehmphul. *Thermal treatment*. https://www.umweltbundesamt.de/en/topics/waste-resources/waste-disposal/thermal-treatment.
(104) Cohen, L. A.; Cavanaugh, T. J.; White, E. *Acrylic/thermoplastic olefin composite*; Google Patents, 2016.
(105) Das, T.; Roy, S. Heat sensing Thermoplastic Elastomer Based on Polyolefins for Encapsulation Applications. *Thermoplastic elastomer-syntesys and application. Cina: Intech* **2015**, 95–113.
(106) Oreski, G.; Ottersböck, B.; Omazic, A. Degradation processes and mechanisms of encapsulants. In *Durability and Reliability of Polymers and Other Materials in Photovoltaic Modules*; Elsevier, 2019; pp 135–152.
(107) Lin, B.; Zheng, C.; Zhu, Q.; Xie, F. A polyolefin encapsulant material designed for photovoltaic modules: from perspectives of peel strength and transmittance. *Journal of Thermal Analysis and Calorimetry* **2020**, *140* (5), 2259–2265.
(108) Barretta, C.; Oreski, G.; Feldbacher, S.; Resch-Fauster, K.; Pantani, R. Comparison of Degradation Behavior of Newly Developed Encapsulation Materials for Photovoltaic Applications under Different Artificial Ageing Tests. *Polymers* **2021**, *13* (2). DOI: 10.3390/polym13020271. Published Online: Jan. 15, 2021.
(109) Adothu, B.; Costa, F. R.; Mallick, S. Damp heat resilient thermoplastic polyolefin encapsulant for photovoltaic module encapsulation. *Solar Energy Materials and Solar Cells* **2021**, *224*, 111024. DOI: 10.1016/j.solmat.2021.111024.
(110) Oreski, G.; Omazic, A.; Eder, G. C.; Voronko, Y.; Neumaier, L.; Mühleisen, W.; Hirschl, C.; Ujvari, G.; Ebner, R.; Edler, M. Properties and degradation behaviour of polyolefin encapsulants for photovoltaic modules. *Prog. Photovolt: Res. Appl.* **2020**, *28* (12), 1277–1288. DOI: 10.1002/pip.3323.

(111) Kempe, M. D.; Nobles, D. L.; Postak, L.; Calderon, J. A. Moisture ingress prediction in polyisobutylene‐based edge seal with molecular sieve desiccant. *Prog. Photovolt: Res. Appl.* **2018**, *26* (2), 93–101. DOI: 10.1002/pip.2947.
(112) Kempe, M. D.; Dameron, A. A.; Moricone, T. J.; Reese, M. O. Evaluation and modeling of edge-seal materials for photovoltaic applications. In *2010 35th IEEE Photovoltaic Specialists Conference (PVSC 2010)*:*Honolulu, Hawaii, USA, 20 - 25 June 2010*; IEEE: Piscataway, NJ, 2010; pp 256–261. DOI: 10.1109/PVSC.2010.5614463.
(113) Reese, M. O.; Dameron, A. A.; Kempe, M. D. Quantitative calcium resistivity based method for accurate and scalable water vapor transmission rate measurement. *The Review of scientific instruments* **2011**, *82* (8), 85101. DOI: 10.1063/1.3606644.
(114) Becker, H.; Brucher, H.; Schott, N.; Rasal, R. *Use of calcium oxide as a water scavenger in solar applications*; Google Patents, 2012.
(115) Cao, J.; Herr, D. E. *Radiation-curable desiccant-filled adhesive/sealant*; Google Patents, 2008.
(116) S. Lassacher, K. Fazeni-Fraisl, and J. Lindorfer. *OPTimized conversion of residual wheat straw to bio-ISObutene for bio based CHEMicals, Grant Agreement n° 744330 - Innovation Action-Demonstration. Project Deliverable D7.1 Full Life Cycle Assessment report of fossil isobutene reference process according to ISO 14040/14044 Document Classification Title Full Life Cycle Assessment report of fossil isobutene reference process according to ISO 14040/14044 Deliverable D7.1 Reporting Period: RP1 Date of Delivery foreseen in DoA."*.
(117) Aoshima, S.; Kanaoka, S. A renaissance in living cationic polymerization. *Chemical reviews* **2009**, *109* (11), 5245–5287. DOI: 10.1021/cr900225g.
(118) Kostjuk, S. V.; Yeong, H. Y.; Voit, B. Cationic polymerization of isobutylene at room temperature. *J. Polym. Sci. A Polym. Chem.* **2013**, *51* (3), 471–486. DOI: 10.1002/pola.26423.
(119) Kempe, M.; Korkmaz, K.; Postak, L.; Booth, D.; Lu, J.; Kotarba, C.; Rupert, L.; Molnar, T.; Aoki, T. Using a butt joint test to evaluate photovoltaic edge seal adhesion. *Energy Science & Engineering* **2019**, *7* (2), 354–360.
(120) Drobny, J. G. 5-Electron Beam Processing of Commercial Polymers, Monomers, and Oligomers. *Ionizing Radiation and Polymers* **2013**, 101–147.
(121) Giacchetta, G.; Leporini, M.; Marchetti, B. Evaluation of the environmental benefits of new high value process for the management of the end of life of thin film photovoltaic modules. *Journal of Cleaner Production* **2013**, *51*, 214–224. DOI: 10.1016/j.jclepro.2013.01.022.
